# Structural and functional specialization of *Bordetella pertussis* DsbA for pertussis toxin folding

**DOI:** 10.1002/pro.70421

**Published:** 2025-12-23

**Authors:** Stephanie Penning, Lachlan Mitchell, Yaoqin Hong, Taylor Cunliffe, Pramod Subedi, Geqing Wang, Lilian Hor, Makrina Totsika, Jason J. Paxman, Begoña Heras

**Affiliations:** ^1^ Department of Biochemistry and Chemistry, La Trobe Institute for Molecular Science, School of Agriculture Biomedicine and Environment La Trobe University Bundoora Australia; ^2^ Centre for Immunology and Infection Control, School of Biomedical Sciences Faculty of Health, Queensland University of Technology Brisbane Australia; ^3^ Present address: Biomedical Sciences and Molecular Biology College of Medicine and Dentistry, James Cook University Townsville Australia; ^4^ Present address: Burnet Diagnostic Initiative, Burnet Institute Melbourne Victoria Australia

**Keywords:** disulphide bond formation, protein folding, structural biology, thioredoxin, virulence factors

## Abstract

Disulphide bonds (Dsbs) are essential for the folding, stability, and function of many secreted and membrane‐associated proteins in bacteria. In Gram‐negative species, these bonds are introduced by the Dsb enzyme family, with DsbA acting as the primary thiol oxidase. While DsbA proteins share a conserved thioredoxin (TRX)‐like fold, emerging evidence highlights substantial structural and functional divergence among pathogenic homologues. Here, we present the high‐resolution crystal structure and functional characterization of BperDsbA, a DsbA homologue from *Bordetella pertussis*, the causative agent of whooping cough. BperDsbA adopts a canonical TRX fold with a CPHC active site and a threonine‐containing cis‐proline loop, but displays striking deviations from prototypical DsbAs. Notably, it contains a highly destabilizing catalytic Dsb, resulting in one of the most oxidizing redox potentials recorded for a DsbA enzyme. Surface electrostatic analysis reveals an unusual distribution of positive and negative charge around the active site, in contrast to the broadly hydrophobic catalytic surfaces of other DsbAs. Functionally, BperDsbA shows limited substrate promiscuity and selectively catalyzes the oxidative folding of a pertussis toxin‐derived peptide, supporting a model of substrate specialization. Together, these findings suggest that BperDsbA has evolved unique redox and structural features to support virulence factor maturation in *B. pertussis*. This work expands our understanding of the mechanistic diversity of DsbA enzymes and highlights their potential as pathogen‐specific targets for anti‐virulence therapeutics.

## 1 INTRODUCTION

As the boundary to the extracellular environment and key mediator of cellular interactions, the bacterial cell envelope harbors myriad proteins central to fitness, survival, dissemination, and pathogenesis (Furniss et al., 2022; Heras et al., 2009). This compartment also serves as the primary site for the folding of disulphide bonds (Dsb) contained within membrane‐associated and secreted virulence factors (Bardwell et al., 1993; Landeta et al., 2018). Similar to eukaryotes, prokaryotes possess dedicated machinery to catalyze the formation of Dsbs (Heras et al., 2009). In Gram‐negative bacteria these molecular machines take the form of the Dsb forming proteins (Dsb)—a family of thioredoxin (TRX)‐like thiol‐disulphide oxidoreductases that harness the oxidizing periplasmic environment to form and maintain Dsbs in newly translocated protein substrates (Heras et al., 2009; Landeta et al., 2018).

The archetype for Dsb catalysis and maintenance in Gram‐negative bacteria comes from *Escherichia coli* K‐12, which features two distinct yet cooperative pathways. In the canonical K‐12 pathway, the primary thiol‐oxidase DsbA (EcDsbA) introduces Dsbs into protein substrates via the transfer of its own Dsb. The reduced EcDsbA cysteines are subsequently re‐oxidized by its cognate membrane‐bound oxidase EcDsbB (Bardwell et al., 1991; Heras et al., 2009; Inaba et al., 2006). An additional pathway is also present for the correction of aberrant disulphides, in which the disulphide isomerase EcDsbC corrects non‐native disulphides and is itself maintained in the reduced, active state by EcDsbD (Landeta et al., 2018; Rietsch et al., 1997; Zapun et al., 1995).

The structural architecture of DsbA enzymes is highly conserved and underpins their function as highly reactive thiol‐oxidases. Their catalytic core adopts a thioredoxin (TRX) fold, defined by the presence of a βαβ‐α‐ββα scaffold, which harbors the catalytic CXXC motif (C_31_P_32_H_33_C_34_ in EcDsbA) and a neighboring *cis*‐proline loop (G_149_V_150_P_151_ in EcDsbA) (Heras et al., 2009; Martin et al., 1993; Shouldice et al., 2011). The βαβ and ββα motifs of the TRX‐core fold in DsbA enzymes are separated by a three‐helical bundle that sits above the catalytic cysteines and two additional bridging helices (Heras et al., 2009; Martin et al., 1993; Shouldice et al., 2011). Beyond this conserved structure, DsbA enzymes frequently display a hydrophobic catalytic surface, a feature thought to increase their affinity for newly translocated, and therefore unfolded or partially folded, protein substrates (Bardwell et al., 1993; Martin et al., 1993). DsbA substrates are structurally and functionally diverse, with many involved in promoting virulence phenotypes (Heras et al., 2009; Santos‐Martin et al., 2021). Consequently, DsbA homologues are directly implicated in the pathogenesis of several clinically important bacterial pathogens including *Bordetella pertussis* (Dutton et al., 2008; Heras et al., 2009; Landeta et al., 2018; Totsika et al., 2018), a highly infectious Gram‐negative coccobacillus and causative agent of whooping cough.

The *B. pertussis* Dsb system mirrors that of canonical *E. coli* with identified DsbA, B and C homologues (Stenson & Weiss, 2002), and bioinformatic analysis suggesting the presence of DsbD and DsbG enzymes. *B. pertussis'* single DsbA (BperDsbA) (Heras et al., 2009) and DsbC (BperDsbC) enzymes have been shown to be essential for both the formation and stabilization of the pertussis toxin (PTX), a major virulence factor in *B. pertussis* infection (Scanlon et al., 2019; Stenson & Weiss, 2002). These enzymes contribute to the formation and maintenance of the toxin's 13 inter and intramolecular Dsbs, which are critical for its structural integrity and function (Stein et al., 1994). Furthermore, BperDsbA is also thought to facilitate the assembly of the pertussis toxin liberation (Ptl) complex, which is required for the secretion of PTX (Stenson & Weiss, 2002). Despite the central role of Dsb proteins in the assembly of major *B. pertussis* virulence factors, the Dsb formation machinery in this pathogen remains poorly characterized.

In this study, we report the high‐resolution crystal structure of *B. pertussis* DsbA (BperDsbA), solved at 1.65 Å, along with comprehensive biochemical and biophysical characterization. BperDsbA adopts the canonical DsbA fold, featuring a TRX‐like domain linked by an α‐helical insertion, closely mirroring the architecture of EcDsbA. However, BperDsbA differs markedly from classical EcDsbA in two key respects: it contains a highly destabilizing Dsb that imparts a strongly oxidizing redox potential, and it presents an electropositive catalytic surface alongside an electronegative peptide‐binding groove, contrasting the predominantly hydrophobic surface properties of EcDsbA. Although these distinctive features result in a more restricted substrate specificity, BperDsbA appears to have evolved distinct structural and redox properties to accommodate a specialized set of substrates critical to *B. pertussis* pathogenesis. Notably, we demonstrate its role in the efficient oxidative folding of pertussis toxin (PTX).

## 2 RESULTS

### 2.1 BperDsbA features a highly reactive disulphide bond

**FIGURE 1:**
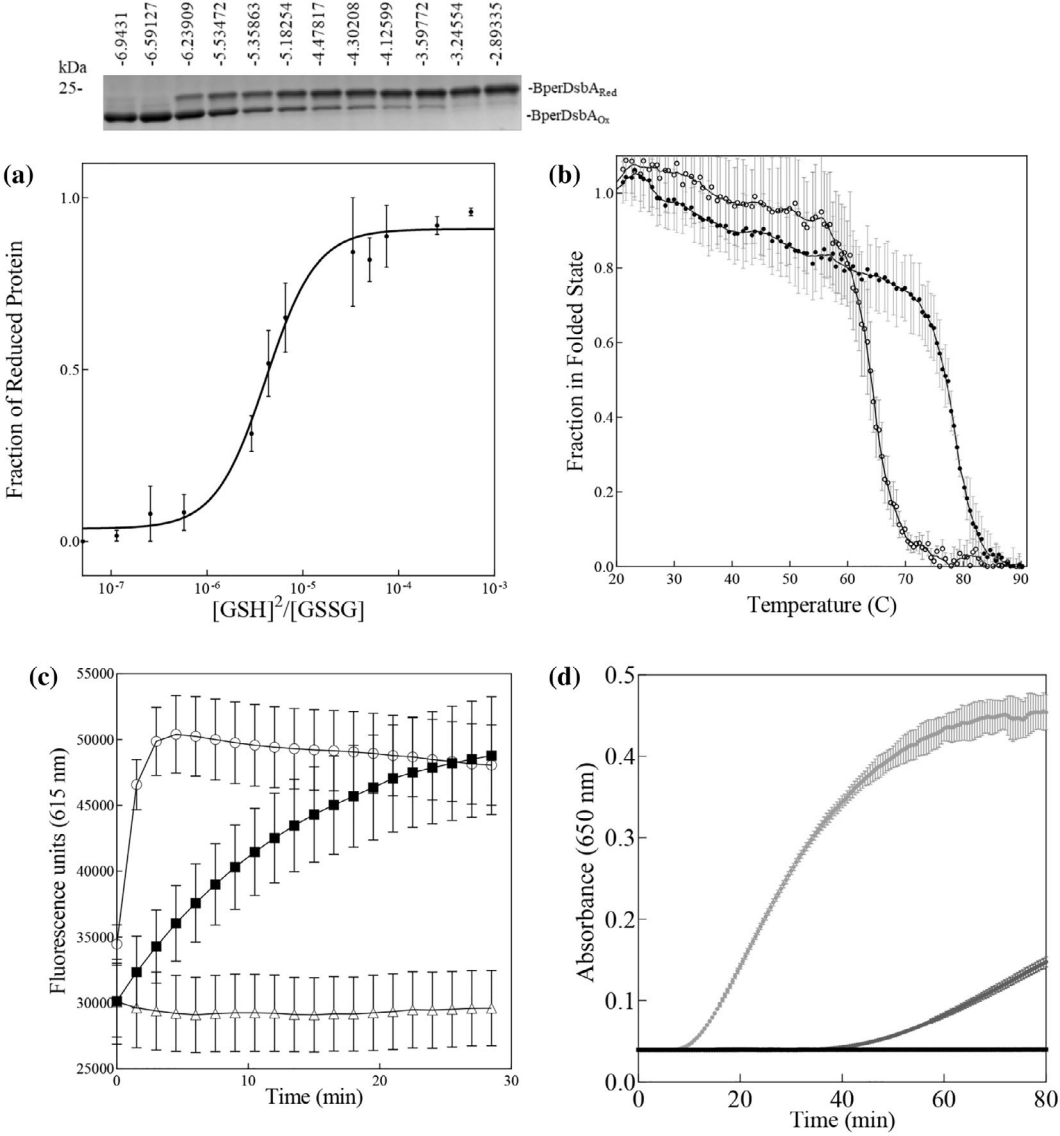
In vitro redox characterization of BperDsbA. (a) The redox potential of BperDsbA. The fraction of reduced protein (representative gel shown above) was plotted against log([GSH]^2^/[GSSG]) to determine the Keq (4.12 ± 1.0 × 10^−6^M) and redox potential was then calculated (−80 mV). Error bars represent SEM. (b) Thermal melt of oxidized (open circles ○) and reduced BperDsbA (closed circles ●). CD thermal melts are plotted as a fraction of α‐helical content (based on molar ellipticity [θ] at 222 nm) and temperature (°C). (c) In vitro thiol oxidase activity. BperDsbA (closed squares ■) shows catalytic activity toward ASST, more comparable to EcDsbA (open circles ○), than to the buffer control (open triangles ∆). Error bars represent SD. (d) In vitro disulphide reductase activity. EcDsbC (light gray ●) and EcDsbA (dark gray ●) showed reductase activity against insulin while neither BperDsbA (black ●) nor a buffer only control (omitted for clarity) showed activity. Error bars represent SD. Statistical analyses for panels (c) and (d) at endpoint are given in Figure S2A, B.

To investigate the oxidizing capacity of BperDsbA, we first determined the protein's redox potential using the glutathione redox couple (GSH/GSSG) as a reference. Redox titration was performed by monitoring AMS alkylation under increasingly reducing conditions. SDS‐PAGE analysis of alkylated samples (representative gel shown in Figure 1a, top panel) followed by densitometric quantification yielded a redox equilibrium constant of 4.12 ± 1.0 × 10^−6^ M, corresponding to an intrinsic redox potential of −80 mV (Figure 1a). This value is significantly more oxidizing than that of EcDsbA (−120 mV) (Inaba & Ito, 2002; Zapun et al., 1993), positioning BperDsbA alongside NmDsbA1 of *Neisseria meningitidis* (−79 mV (Vivian et al., 2009)) as among the most oxidizing DsbA enzymes characterized to date, second only to PaDsbA2 from *Pseudomonas aeruginosa* (−67 mV (Arts et al., 2013; Shouldice et al., 2009; Vivian et al., 2008)).

The highly oxidizing activity of DsbA enzymes often relies on the relative instability of their active‐site Dsb, which facilitates disulphide donation to substrate proteins. To assess the stability of the BperDsbA catalytic disulphide, we used circular dichroism (CD) spectroscopy to compare the thermal stability of the oxidized and reduced forms of the protein. The melting temperature (Tm) of reduced BperDsbA was 78.0°C (±0.3°C), while the oxidized form had a significantly lower Tm of 62.0°C (±0.6°C), a 16°C difference (Figure 1b). This destabilization upon oxidation is consistent with other DsbAs, such as EcDsbA, which exhibits a ΔTm of approximately 9°C (Christensen et al., 2016; Zapun et al., 1993), though the effect in BperDsbA is notably greater. These data suggest that BperDsbA harbors an unusually reactive Dsb and may function as a highly oxidizing enzyme.

To determine whether BperDsbA's unusually reactive and thermodynamically unstable Dsb supports functional thiol‐oxidase activity, we assessed its ability to catalyze disulphide formation in vitro. Thiol‐oxidase activity was first assessed using two established peptide substrates, one derived from an EcDsbL substrate (ASST [**C**NENGL**C**K] (Lee et al., 2004)), and another a Neisserial DsbA substrate (PilQ [**C**QQGFDGTQNS**C**K] (Vivian et al., 2008)). BperDsbA catalyzed disulphide formation in the ASST‐derived peptide, albeit less efficiently than EcDsbA (Figure 1c). In contrast, BperDsbA showed no measurable activity toward the PilQ‐derived peptide compared to EcDsbA which efficiently catalyzed thiol oxidation on this peptide (Figure S1, Supporting Information), indicating substrate specificity.

To further define the redox properties of BperDsbA in vitro, its disulphide‐reductase activity was measured using the standard insulin reduction assay. Unlike EcDsbA, which exhibits some reductase activity (Bardwell et al., 1991), BperDsbA showed no measurable activity against insulin (Figure 1d).

### 2.2 BperDsbA is a functional thiol‐oxidase that exhibits substrate specificity

To investigate the oxidase activity of BperDsbA in vivo, we expressed the protein in EcDsbA‐deficient *E. coli* strains (JCB817 (Bardwell et al., 1991) and PL263 (Leverrier et al., 2011)) and assessed its ability to complement EcDsbA. To confirm correct localization for functional assays, an additional construct encoding a C‐terminal hexahistidine‐tagged BperDsbA was analyzed. Immunoblotting confirmed that BperDsbA is secreted to the periplasm (Figure S3). AMS alkylation of periplasmic extracts further demonstrated that BperDsbA is predominantly present in the oxidized form, indicating that it is correctly processed and maintained in its oxidized, active state, most likely through oxidation by EcDsbB.

**FIGURE 2:**
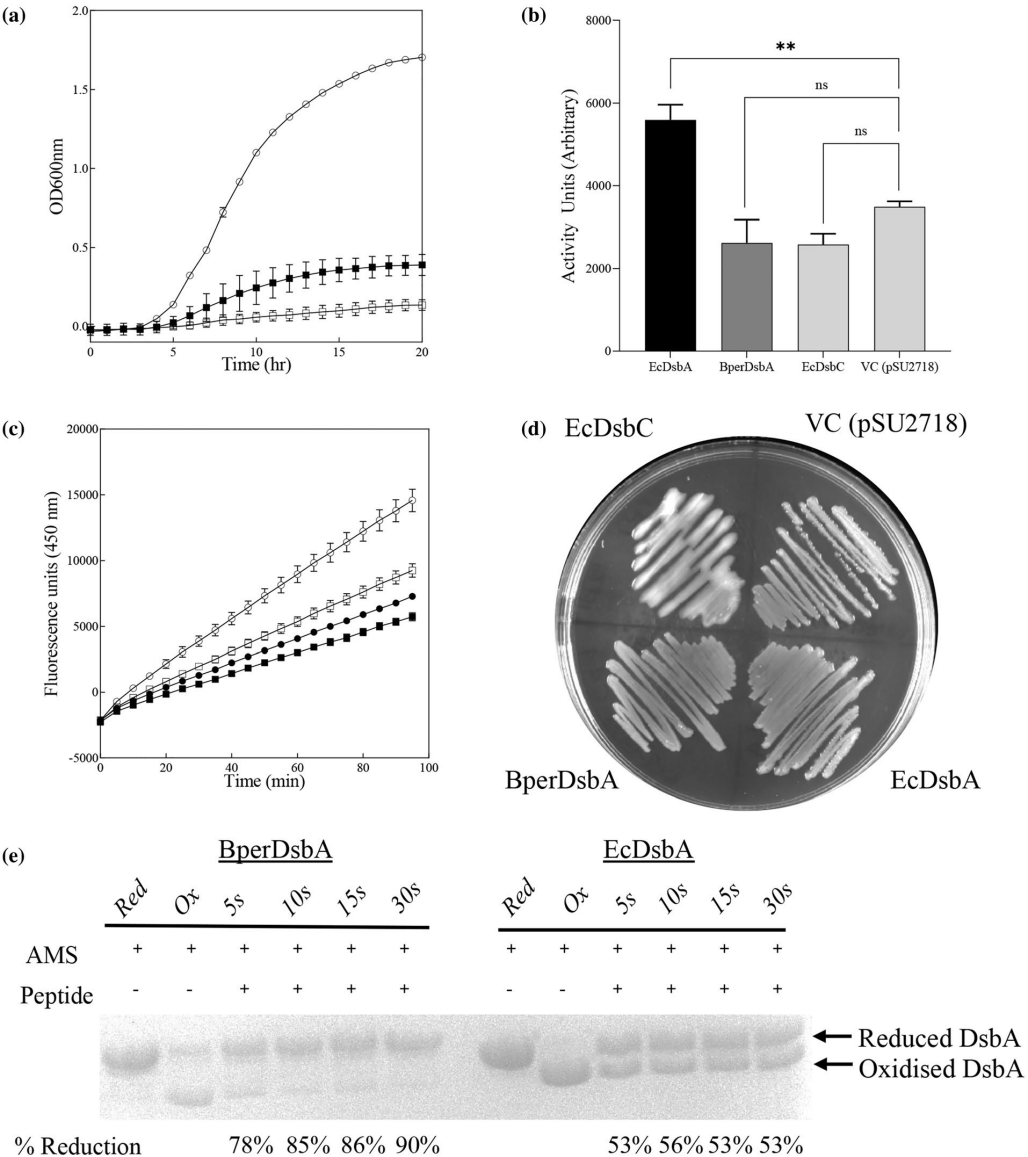
BperDsbA displays a narrow substrate specificity. (a) Swimming motility assay. EcDsbA transformed cells (open circles ○) displayed flagella mediated motility while cells containing BperDsbA (closed squares ■) did not migrate as far. However, BperDsbA did display more motility than the empty vector control (open squares □). (b) In vivo phosphatase activity. Mean activity units of phosphatase activity indicate correct folding of the enzyme PhoA by EcDsbA (black bar), but not by BperDsbA (gray bar) or an empty vector control (light gray bar). The effect of BperDsbA expression in *E. coli* to complement the loss of native EcDsbA was compared using one‐way ANOVA with a Dunnett post‐hoc using VC as the control. Both EcDsbC and BpDsbA are non‐significant compared to the VC (adjusted *p* values are 0.2362 respectively; *p* > 0.05). In contrast, EcDsbA expression, as expected, leads to significant difference compared to the VC (*p* value is 0.0041, i.e., *p* < 0.005 or **). Error bars represent SEM. (c) In vivo ASST oxidase activity. Only EcDsbA (open circles ○) displayed ASST oxidation while BperDsbA (closed squares ■), EcDsbC (closed circles ●) and the vector control (open squares □) all displayed similarly low levels of activity. (d) Mucoidal colony morphology assay. Cells expressing EcDsbC display mucoidal colonies, indicating in vivo isomerase activity while neither BperDsbA nor EcDsbA transformed cells show phenotypic differences compared to an empty vector control. (e) In vitro oxidase activity against native substrate. BperDsbA reacts rapidly with the pertussis toxin (PTX) peptide, reaching 90% reduction within 30 s, whereas EcDsbA reaches only approximately 50% reduction in the same timeframe. The fraction of reduced enzyme was quantified by densitometry using the ImageJ package (Schneider et al., 2012). Statistical analyses for panels (a) and (c) at endpoint are given in Figure S2C, D.

Having established that BperDsbA is correctly expressed in the *E. coli* periplasm and maintained in its oxidized, active form, we next examined its ability to support oxidative protein folding in vivo. Interestingly, BperDsbA only partially restored the oxidative folding of FlgI, a component of the bacterial flagellar motor (Hizukuri et al., 2006; Verderosa et al., 2021) as evidenced by swimming motility (Figure 2a), indicating that it possesses thiol‐oxidase activity in vivo. However, it failed to restore the activity of the alkaline phosphatase PhoA (Figure 2b), with phosphatase activity in cells expressing BperDsbA comparable to the empty vector. Unlike EcDsbA, BperDsbA also failed to catalyze the folding of the full‐length EcDsbL substrate ASST in cell‐based assays (Figure 2c), despite being able to oxidize an ASST‐derived peptide (Figure 1c). Similarly to EcDsbA, BperDsbA failed to function as a disulphide isomerase in vivo (Figure 2d), with transformant colonies showing no change in mucoidal morphology dependent on the isomerisation of RcsF (Leverrier et al., 2011).

Together, these findings suggest that BperDsbA is a functional oxidase but displays a narrow substrate specificity. To further probe substrate recognition, we tested BperDsbA's activity against a synthetic peptide derived from the *B. pertussis* toxin PTX (QI**C**PLNGY**C**E), a known physiological target (Stenson & Weiss, 2002). This was achieved by monitoring the formation of reduced DsbA (both BperDsbA and an EcDsbA control) using AMS alkylation in the presence of the PTX peptide, indicative of their disulphide transfer. BperDsbA efficiently catalyzed the oxidative folding of this peptide and, notably, did so more efficiently than EcDsbA (Figure 2e, Figure S4A). A second PTX‐derived peptide (SI**C**NPGSSL**C**) was also tested in the same manner. Neither BperDsbA nor EcDsbA was fully reactive toward this second PTX peptide in the timeframe tested; however, a greater proportion of BperDsbA became reduced compared to EcDsbA during the same reaction time (Figure S4B). This confirms that BperDsbA is a competent thiol‐oxidase with efficient catalytic activity when acting on select substrates.

### 2.3 BperDsbA shares the canonical DsbA architecture

We determined the crystal structure of BperDsbA to identify any structural features that might influence the instability of the catalytic disulphide and its substrate specificity. The structure of BperDsbA was solved to a resolution of 1.65 Å using molecular replacement and refined to R‐factor and R‐free values of 16.87% and 20.16%, respectively (Table 1). The asymmetric unit contains two symmetrically independent monomers, both encompassing residues 1–179, which superimpose with a root‐mean‐square deviation (RMSD) of 0.432 Å across 179 equivalent Cα atoms (Figure S5). Given the structural similarity between both monomers, monomer A was used for further analysis.

**TABLE 1:** Data collection and refinement statistics.

PDB ID: 9PH2	
Data collection	
Resolution range(Å)	40.98–1.65 (1.68–1.65)
Wavelength (Å)	0.5373
Space group	*C*2
Unit cell parameters (Å, °)	*a* = 191.785, *b* = 37.298, *c* = 57.111, *α* = *γ* = 90, *β* = 104.593
Number of molecules per asymmetric unit	2
Total number of reflections	195,036
Number of unique reflections	46,464 (2422)
Completeness (%)	97.53 (86.44)
Redundancy	4.3 (3.9)
I/σ (I)	9.5 (2.9)
Rmerge	0.064 (0.301)
Rpim	0.037 (0.186)
Rmeas	0.079 (0.380)
Refinement	
Resolution (Å)	40.98–1.65 (1.68–1.65)
*R*work	16.87 (20.17)
*R*free	20.16 (22.67)
CCwork	0.919 (0.889)
CCfree	0.881 (0.874)
No. of atoms (excluding H)	
Protein	2872
Solvent	539
PEG	7
Protein residues	358
R.M.S.D from ideal geometry	
Bonds (Å)	0.005
Angles (°)	0.79
Ramachandran (%)	
Favored	99.72
Allowed	0.28
Outliers	0.00
Rotamer outliers (%)	0.00
Clashscore (%)	1.22
Average B‐factors	
Protein	18.12
Solvent	32.04
PEG	40.01

*Note*: Values in parentheses represent the highest resolution shell.

**FIGURE 3:**
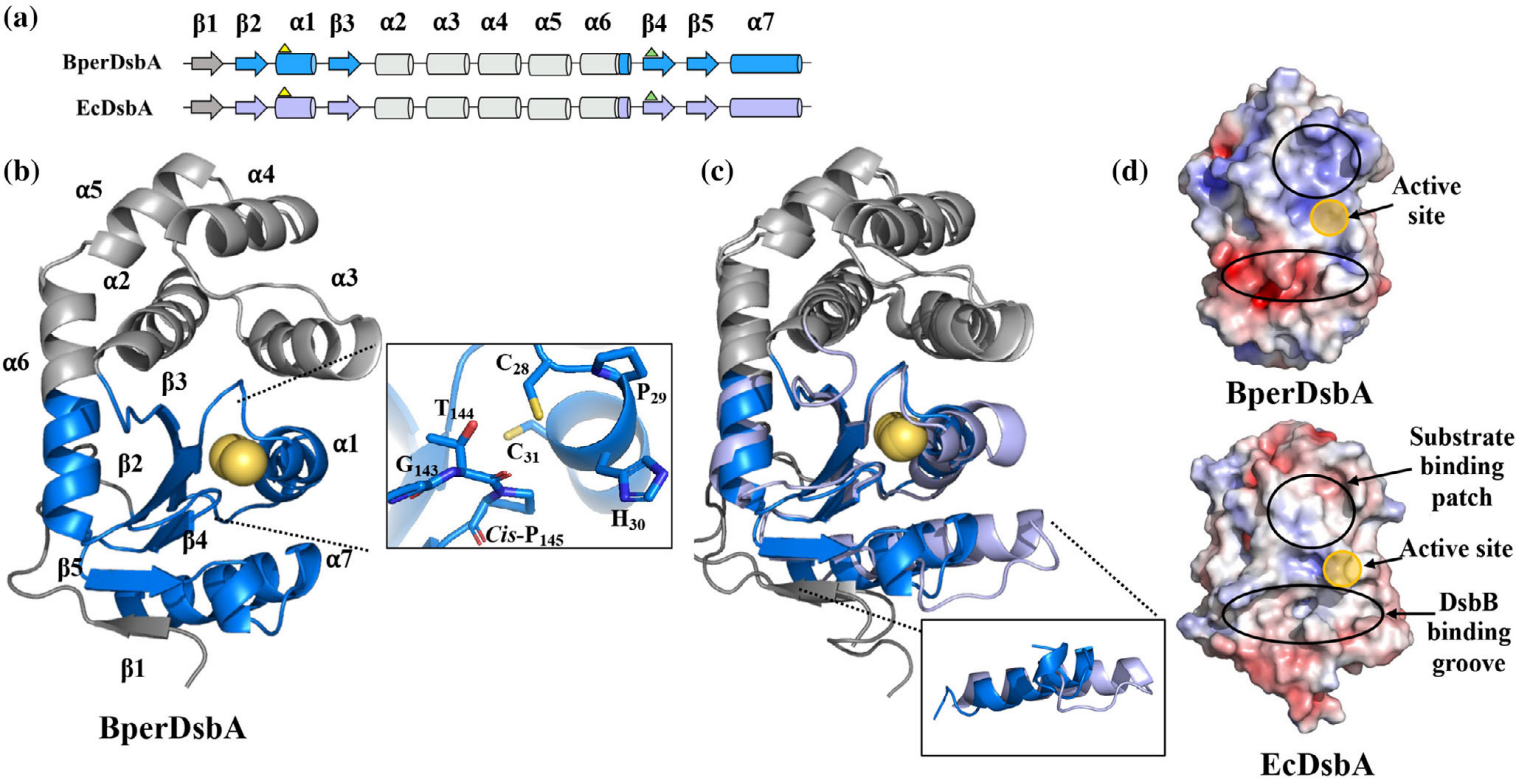
Structural comparison of BperDsbA to EcDsbA. (a) Domain organization of BperDsbA and EcDsbA. BperDsbA shares the conserved DsbA domain organization evidenced by the TRX‐like βαβ and ββα motifs (blue and purple for BperDsbA and EcDsbA, respectively) separated by an insertion of five helices (white). A short N‐terminus (gray) is present in both sequences. (b) Cartoon depiction of BperDsbA. Secondary structural elements are numbered from N to C terminus and colored as per (a). Catalytic cysteines are shown as yellow spheres. The inset shows a stick depiction of the BperDsbA active site encompassing the CPHC redox centre and GT*cis*P loop). (c) Superimposition of BperDsbA and EcDsbA. BperDsbA (blue) superimposes onto the structure of EcDsbA (chain A PDB ID: 1FVK (Guddat et al., 1997; Martin et al., 1993)) (purple) with an RMSD of 2.1 Å across 161 equivalent Cα atoms with the most significant structural difference evident in the topology of the loop connecting β_5_ and α_7_ (cutout). (d) Electrostatic surface representation of BperDsbA (top) and EcDsbA (PDB ID: 1FVK (Guddat et al., 1997; Martin et al., 1993)) (bottom). BperDsbA possesses the canonical surface features of DsbA enzymes with an identifiable substrate binding patch and DsbB binding groove however these features in BperDsbA carry an unusual positive and negative charge, respectively. The electrostatic potential was calculated with APBS (Baker et al., 2001) in PyMOL (Delano, 2002) showing positive charges in blue (saturating at 5 kT/e) and negative charges in red (saturating at −5 kT/e).

BperDsbA shares the canonical DsbA‐like architecture (Martin et al., 1993) with a TRX‐like core fold defined by the conserved motifs β₂α₁β₃ (residues 19–63) and β₄β₅α₇ (residues 130–179) separated by an alpha helical insertion domain (Figure 3a,b). This insertion includes a three‐helix bundle (α₂, α₃, α₄; residues 64–111) and two additional helices (α₅ and α₆; residues 112–129). The active site includes conserved catalytic residues, with the CXXC motif (C_28_P_29_H_30_C_31_) located at the tip of α₁ (Quan et al., 2007) and the proline loop (G_142_T_143_P_144_) bridging α_6_ and β_4_ (Figure 3b, inset) (Ren et al., 2009).

Structural superimposition of BperDsbA with the reference protein EcDsbA (PDB ID: 1FVK chain A (Guddat et al., 1997)), shows that despite their moderate sequence identity of 25%, the two proteins share considerable structural similarity, aligning with a RMSD of 2.1 Å across 161 equivalent Cα atoms (Figure 3c and S6). The most notable structural difference lies in the conformation of the loop spanning β₅ and α₇ (Figure 3c, inset), which affects the positioning of helix α₇. Although this loop contains a similar number of residues in both proteins (residues 184–193 and 162–170 in BperDsbA and EcDsbA, respectively), BperDsbA incorporates a three‐residue half‐helical turn that effectively shortens the loop and repositions the N‐terminal end of α₇ closer to the catalytic core (Figure 3b).

Consistent with their moderate sequence identity, BperDsbA and EcDsbA exhibit distinct surface electrostatic properties, particularly in the regions surrounding their catalytic sites, which are critical for redox activity. BperDsbA features an overall electropositive surface near the catalytic cysteines, including a prominent basic patch located directly above the active site (Figure 3d, top panel). In contrast, the corresponding region in EcDsbA, previously described as the substrate‐binding patch (Paxman et al., 2009), is predominantly hydrophobic (Figure 3d, bottom panel). Additionally, the groove located below the active site is significantly more acidic in BperDsbA compared to EcDsbA. In EcDsbA, this groove has been characterized as the DsbB‐binding site (Inaba et al., 2006). These differences in surface properties suggest divergent mechanisms of substrate and partner recognition between the two proteins.

Finally, BperDsbA features an acidic cavity on the non‐catalytic face, opposite the active site, which forms the entrance to a buried water channel, an element predicted to be conserved across DsbA proteins (Figure S7) (Wang et al., 2023). This polar channel connects the catalytic cysteines to the bulk solvent through a network of hydrogen‐bonded water molecules, which has been shown to be important for catalytic activity. In BperDsbA, the channel is coordinated by Cys_31_, Glu_22_, Glu_35_, and Gln_53_, along with two ordered waters. A comparable arrangement is observed in EcDsbA, where the equivalent residues are Cys_33_, Glu_24_, Glu_37_, and Lys_58_ (Figure S7).

### 2.4 Structural features of the BperDsbA may underlie its highly oxidizing redox potential

As the chemical environment surrounding the catalytic center plays a key role in regulating redox activity, the structure of this region in BperDsbA was analyzed to identify features that may contribute to its highly oxidizing nature. The residues forming the catalytic dipeptide—positioned between the two catalytic cysteines—are known to influence the redox potential of DsbA proteins (Huber‐Wunderlich & Glockshuber, 1998; Quan et al., 2007). BperDsbA possesses a CPHC active site, one of the most common motifs among DsbA homologues, with proline and histidine found at the dipeptide positions in approximately 76% and 79% of homologues, respectively (Quan et al., 2007). Although the protein was oxidized prior to crystallization, the structure revealed BperDsbA in a reduced form, likely due to radiation damage.

**FIGURE 4:**
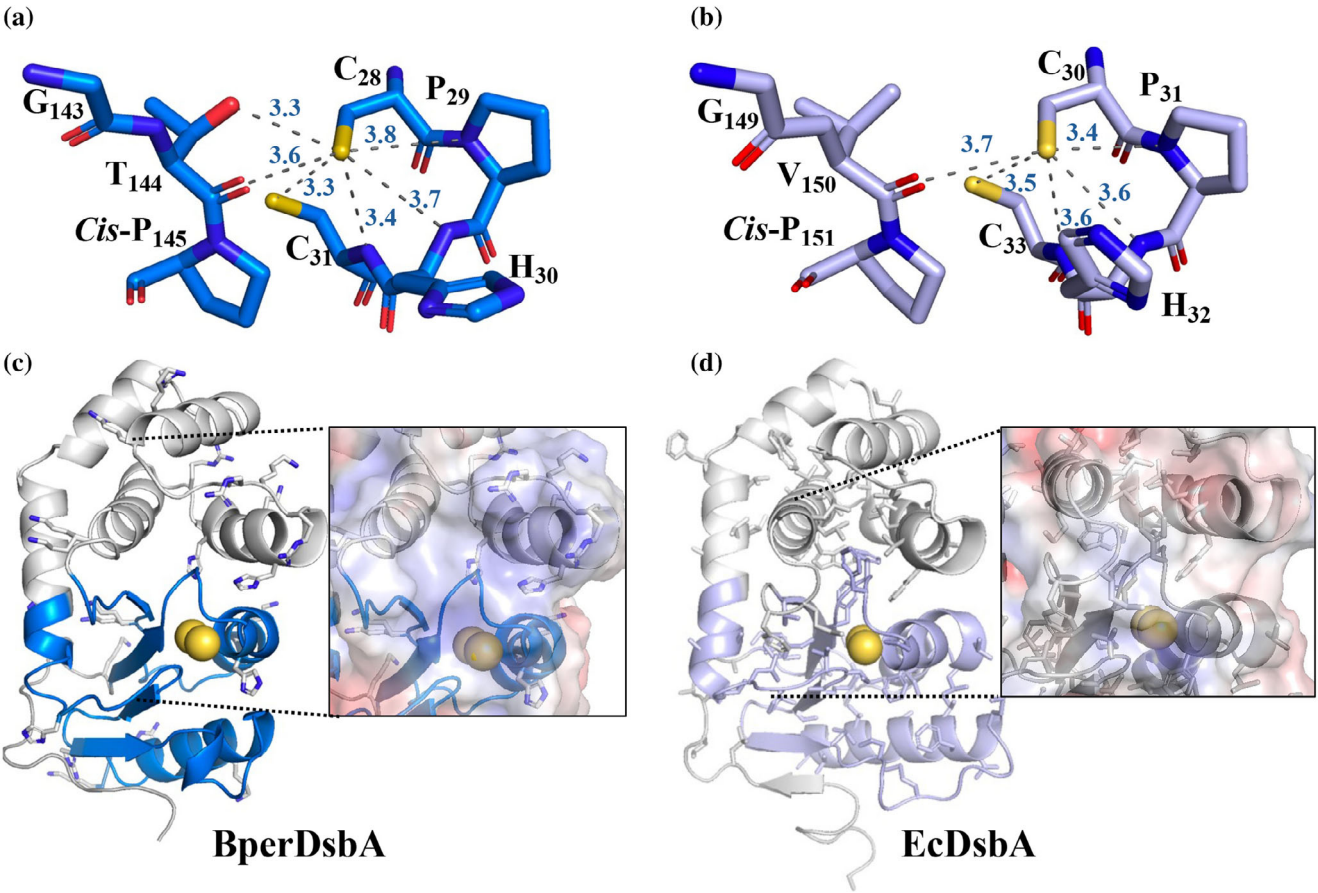
Structural features of BperDsbA contributing to its highly oxidizing redox potential. (a) Active site of reduced BperDsbA showing hydrogen‐bond interactions stabilizing the thiol form of the N‐terminal catalytic cysteine (Cys_28_) within the CPHC motif. The sulfur atom of Cys_28_ forms hydrogen bonds (dotted lines) with the main‐chain nitrogen atoms of Pro_29_, His_30_, and Cys_31_, as well as with both the side‐chain hydroxyl and main‐chain carbonyl of Thr_144_ from the cis‐proline loop (G_142_T_143_P_144_). (b) Active site of reduced EcDsbA (PDB ID: 1A2L (Guddat et al., 1998) showing similar intra‐CPHC hydrogen bonds but lacking the additional interaction from a hydroxyl‐bearing side chain in the *cis*‐proline loop, due to the presence of Val_150_. (c) Cartoon representation of BperDsbA highlighting positively charged residues. Clusters of positive residues clustered around the active site, generate an electropositive patch that may stabilize the Cys_28_ thiolate (inset). (d) Cartoon representation of EcDsbA showing a predominantly hydrophobic environment near the active site (inset), lacking the electropositive character observed in BperDsbA.

In this reduced state, hydrogen‐bond contacts were observed between the sulfur atom of Cys_28_ and the main‐chain nitrogen atoms of Pro_29_, His_30_, and Cys_31_ within the CPHC motif (Figure 4a). Additionally, Cys_28_ forms hydrogen bonds with both the main‐chain carbonyl and side‐chain hydroxyl group of Thr_144_, a residue located in the *cis*‐proline loop (G_142_T_143_P_144_). EcDsbA is known to exhibit similar interactions around its equivalent catalytic cysteine (Cys_30_) when both oxidized and reduced (Guddat et al., 1998) however, EcDsbA contains a valine residue preceding the *cis*‐proline loop, where BperDsbA features threonine. Hydrogen‐bonding interactions from this position are known to stabilize the reduced state and elevate the redox potential in thioredoxin‐like proteins (Ren et al., 2009). This valine is capable of forming hydrogen bonds only through its main‐chain carbonyl, lacking the additional stabilizing bond provided by a side‐chain hydroxyl group (Figure 4b); thus likely contributing to the difference in redox potential between the two enzymes.

Beyond hydrogen bonding and the conserved CPHC motif, the electrostatic environment surrounding the BperDsbA active site may further contribute to its strong oxidizing capability. Several positively charged residues are clustered on the catalytic surface of the protein (Figure 4c), likely stabilizing the thiolate form of Cys_28_. This electrostatic stabilization lowers the pKₐ of the thiol group, enhancing nucleophilicity and thereby increasing the redox potential of the protein (measured at −80 mV). In contrast, EcDsbA, which exhibits a lower redox potential (−120 mV), has a predominantly hydrophobic environment surrounding its active site, offering less electrostatic stabilization of the thiolate.

Taken together, the specific dipeptide composition of the CPHC motif, the *cis*‐proline loop‐mediated hydrogen‐bonding network, and the electropositive character of the active‐site surface act in concert to fine‐tune BperDsbA's redox activity and underpin its heightened oxidizing power.

### 2.5 BperDsbA features a class Ib type topology

**FIGURE 5:**
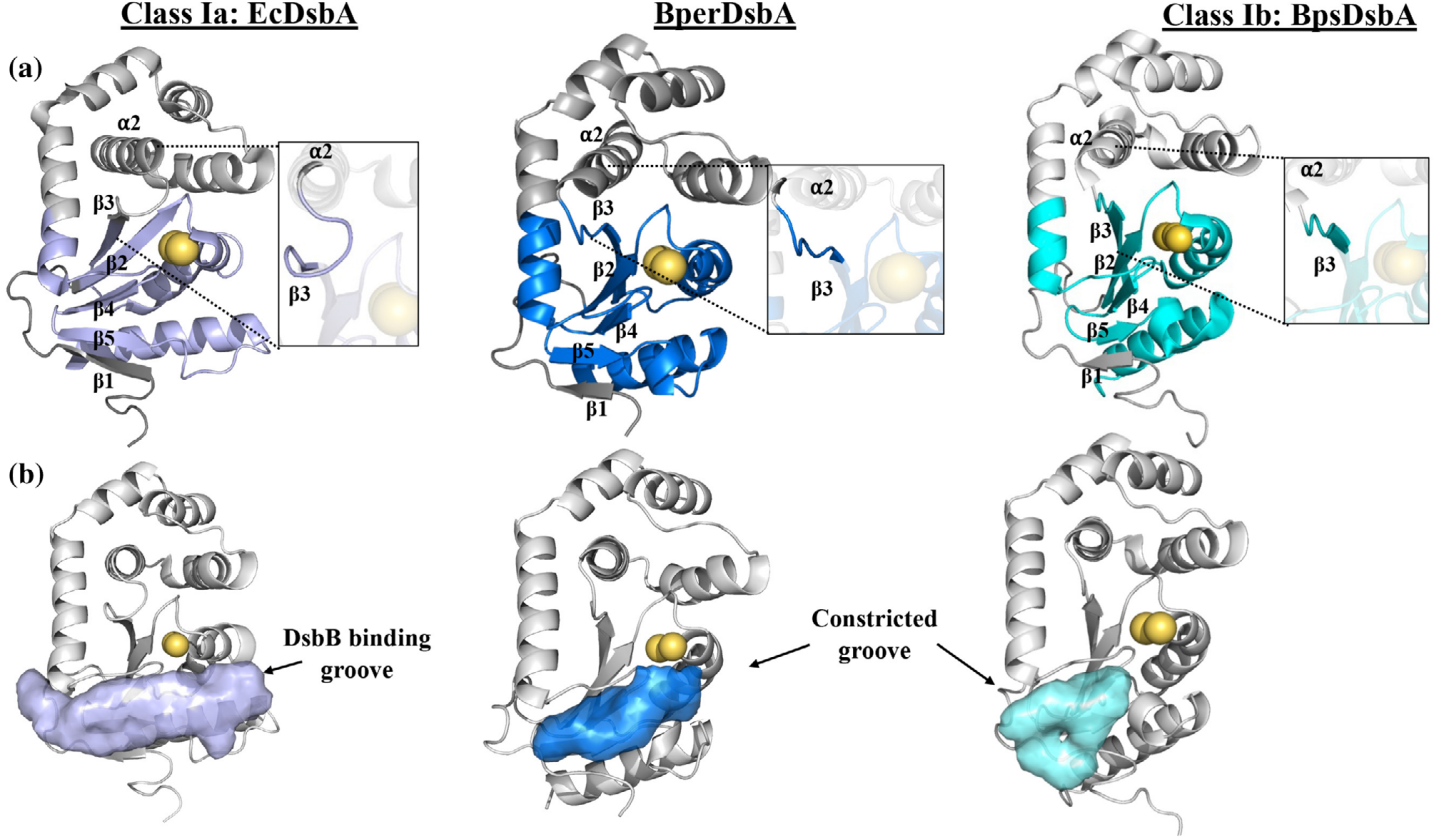
BperDsbA shares features of Class Ib DsbA architecture. (a) Structural comparison of, from left to right, EcDsbA (PDB ID: 1FVK (Guddat et al., 1997; Martin et al., 1993)), BperDsbA (blue), and BpsDsbA (PDB ID: 42KD (Ireland et al., 2013)). The topology of the β_6_‐α_2_ loop (inset) differs between class Ia and Ib DsbAs, with the orientation of this loop pointed toward the catalytic cysteines in class Ia members, typified by EcDsbA, and away from it in class Ib members, typified by BpsDsbA. The BperDsbA loop mirrors that of BpsDsbA, supporting a class Ib topology. (b) Surface depictions of the DsbB binding groove of, from left to right, EcDsbA, BperDsbA and BpsDsbA. The DsbB binding groove (indicated by colored region) is bound by the *cis‐*proline loop and the β₅ and α₇ loop. In class Ia DsbAs, like EcDsbA, this groove is long‐ extending past the catalytic cysteines, while in class Ib DsbAs, like BpsDsbA, this region is truncated due to the short helical turn in the β₅ and α₇ loop. This equivalent region in BperDsbA is narrower than in EcDsbA and mimics the topology seen in class Ib DsbAs.

To better understand the structural basis for BperDsbA's substrate specificity, we compared its topology with other structurally characterized DsbA proteins. DsbAs are broadly categorized into two major structural classes based on the arrangement of β‐strands within their TRX domain. In class I DsbAs, such as EcDsbA, the β₁ strand at the N‐terminus forms hydrogen bonds with β₅, producing a core β‐sheet topology of 3‐2‐4‐5‐1 (Figure 5a, left). Conversely, class II DsbAs feature β₁ hydrogen bonding to β₃, leading to a topology of 1‐3‐2‐4‐5 (Figure S8) (McMahon et al., 2014).

The β‐sheet topology of BperDsbA matches that of class I DsbAs: β₁ hydrogen bonds to β₅, forming a 3‐2‐4‐5‐1 pattern (Figure 5a, middle). However, a closer examination reveals that BperDsbA belongs specifically to the class Ib subclass. This classification is based on conformational differences in loop regions that surround the catalytic site, particularly the loop between β₂ and α₂, which connects the TRX domain to the helical domain. In class Ia DsbAs (e.g., EcDsbA), this loop projects inward, toward the active site (Figure 5a, left). In contrast, class Ib DsbAs, exemplified by *Burkholderia pseudomallei* BpsDsbA (PDB ID: 4K2D (McMahon et al., 2014)), exhibit an outward‐facing β₂–α₂ loop conformation (Figure 5a, right). BperDsbA mirrors this class Ib configuration, with the corresponding loop oriented away from the active site, further supporting its classification within this subclass.

Another hallmark of class Ib DsbAs is the presence of a truncated and less well‐defined hydrophobic groove. This feature arises from structural alterations in the β₅ strand, the α₇ helix, and the intervening β₅–α₇ loop. BperDsbA follows this architecture; it exhibits a conformationally distinct β₅–α₇ loop, along with a shortened α₇ helix—reduced by approximately two helical turns relative to EcDsbA (Figure 5a, left vs. middle). These structural deviations reshape the β₅–β₆–α₇ region, which contributes one edge of the canonical DsbB peptide‐binding groove in DsbA proteins. In BperDsbA, this motif forms a more compact and constricted groove (Figure 5b, middle), in contrast to the broader, more accessible substrate‐binding cleft observed in EcDsbA (Figure 5b, left). This narrower groove may impose steric constraints on substrate engagement, contributing to BperDsbA's apparent substrate selectivity.

Further supporting its classification as a Class Ib DsbA, structural homology analysis using the DALI server (Holm et al., 2023) identified BperDsbA's closest structural analogues as other Class Ib DsbAs. Notably, *P. aeruginosa* PaDsbA1 (PDB ID: 3H93 (Shouldice et al., 2009)) shares 38% sequence identity with BperDsbA and aligns with a root mean square deviation (RMSD) of 1.3 Å across 177 equivalent Cα atoms. Similarly, *B. pseudomallei* BpsDsbA shows 39% sequence identity and an RMSD of 1.4 Å over the same number of aligned residues (Figure S6). These structural parallels reinforce the classification of BperDsbA as a class Ib thiol‐disulfide oxidoreductase.

Together, these structural features firmly position BperDsbA within the class Ib subclass of DsbA enzymes and offer insight into its unique substrate recognition profile. The narrow and more spatially restricted peptide binding groove adjacent to the CPHC active site, likely imposes steric constraints that limit accommodation of substrate. Furthermore, BperDsbA exhibits an unusual surface electrostatic distribution, characterized by a positive patch directly above the active site and a negatively charged groove below. This charge distribution may enhance selectivity by favoring substrates with complementary surface features.

## 3 DISCUSSION

*B. pertussis* is a Gram‐negative coccobacillus and the causative agent of whooping cough, a highly contagious respiratory disease that remains a leading cause of death among children under the age of 10 globally (Tan et al., 2015; Vos et al., 2020). The continued relevance of this disease is driven by the emergence of *B. pertussis* strains that evade immunity conferred by current acellular vaccines (Ma et al., 2021) and the increasing resistance to macrolide antibiotics—currently the treatment of choice for *B. pertussis* infections (Ivaska et al., 2022). These limitations highlight the urgent need for new therapeutic strategies, including those that target bacterial virulence pathways.

One such target is the bacterial Dsb system, particularly the DsbA enzymes that catalyze Dsb formation in substrates associated with bacterial fitness and pathogenesis (Bardwell et al., 1993; Furniss et al., 2022; Heras et al., 2009; Kadokura et al., 2004; Silverman et al., 1968). Although these enzymes share a conserved structural framework, they exhibit distinct redox properties and surface features tailored to the folding of species‐specific substrates, many of which contribute directly to virulence [4, 2, 11, 47]. In *B. pertussis*, BperDsbA is essential for the folding of the pertussis toxin (PTX), a key virulence determinant (Smith et al., 2001; Stenson & Weiss, 2002), making it a compelling target for anti‐virulence drug development (Abulaila et al., 2024; Adams et al., 2015; Duncan et al., 2021; Heras et al., 2015; Landeta et al., 2018; Smith et al., 2016; Wang et al., 2022).

Here, we provide the first comprehensive structural and functional characterization of BperDsbA. Our data show that this protein is a structurally canonical but functionally specialized member of the DsbA family. While Dsbs generally confer structural stability to proteins, the catalytic Dsbs of DsbAs are generally destabilizing, which makes reduction thermodynamically favorable and contributes to the highly oxidizing redox potentials of these enzymes (Wunderlich & Glockshuber, 1993; Zapun et al., 1993). Biochemical analysis revealed that BperDsbA harbors a highly destabilizing catalytic Dsb, with a ΔTm of 16°C between its reduced and oxidized forms, substantially higher than the 8–12°C typical of other DsbAs (Wunderlich & Glockshuber, 1993; Zapun et al., 1993), and comparable only to NmDsbA1 from *N. meningitidis* (Lafaye et al., 2009). Notably, BperDsbA, along with NmDsbA1, ranks among the most oxidizing DsbAs characterized to date, with redox potentials of −80 and −79 mV (Vivian et al., 2009) respectively, second only to PaDsbA2 of *P. aeruginosa* (−67 mV (Arts et al., 2013; Shouldice et al., 2009; Vivian et al., 2008)).

Highly oxidizing redox potentials and destabilized Dsbs in DsbAs, facilitate the efficient transfer of Dsbs to substrate proteins (Bardwell et al., 1991). However, despite its strong oxidative potential, BperDsbA exhibits limited substrate promiscuity. When tested in DsbA‐deficient *E. coli* strains, BperDsbA was unable to fully restore the activity of FlgI and was unable to oxidize PhoA or full‐length ASST. In vitro BperDsbA failed to oxidize a peptide derived from a Neisserial protein (PilQ) and inefficiently interacted with a peptide derived from *E. coli* ASST (Lee et al., 2004). However, BperDsbA efficiently oxidized two PTX‐derived peptides, suggesting that BperDsbA's restricted activity reflects a finely tuned substrate specificity, rather than a lack of oxidizing capacity.

To contextualize these properties, we determined the 1.65 Å crystal structure of BperDsbA. The protein adopts a conserved DsbA‐like architecture (Shouldice et al., 2011) with loop topologies consistent with a class Ib classification (McMahon et al., 2014), and revealed several structural features that likely contribute to the highly oxidizing redox potential of BperDsbA. Firstly, BperDsbA contains a CPHC active‐site motif—one of the most common catalytic sequences in DsbA homologues (Heras et al., 2009) and associated with their oxidizing potential (Huber‐Wunderlich & Glockshuber, 1998; Quan et al., 2007).

Secondly, an additional hydrogen bond network stabilizes the thiol/thiolate form of Cys_28_. In regard to the DsbAs containing a CPHC catalytic site, those that have a valine preceding the *cis*‐proline residue, like EcDsbA (Heras et al., 2010; Kurth et al., 2013; Martin et al., 1993; Walden et al., 2012), generally feature a less oxidizing redox potential than those with threonine, likely because valine is unable to engage in as many stabilizing hydrogen bonds. However, exceptions exist, as PaDsbA1 of *P. aeruginosa* (Shouldice et al., 2009) and BpsDsbA of *B. pseudomallei* (Ireland et al., 2013) remain highly oxidizing (both −94 mV) despite harboring a valine at this position.

A third contributing feature is the presence of a positively charged electrostatic patch surrounding the active site, which likely further stabilizes the thiolate anion of Cys_28_ (McMahon et al., 2014; Nelson & Creighton, 1994). A similar stabilizing mechanism has been proposed for DsbL, where a cluster of basic residues surrounding the active site supports highly oxidizing redox potentials (−95 mV for EcDsbL and −97 mV for SeDsbL) (Grimshaw et al., 2008; Heras et al., 2010), although BperDsbA is not as electropositive. These combined elements—a CPHC motif, an extensive hydrogen‐bonding network, and a basic electrostatic microenvironment—likely fine‐tune the enzyme's redox potential.

BperDsbA also exhibits notable structural differences compared to class Ia DsbAs such as EcDsbA, including a restructured β5–α7 loop that produces a narrower peptide‐binding groove more consistent with class Ib DsbAs (McMahon et al., 2014; Santos‐Martin et al., 2021) and distinct surface electrostatics. These features, likely underlie its restricted substrate range and adaptation to specific PTX‐related targets. Indeed, variations in the β5–α7 region among DsbA homologues have been associated with changes in the shape and chemical properties of the peptide‐binding groove, as well as differences in substrate selectivity. In particular, class Ib enzymes that diverge from the EcDsbA β5–α7 topology often display narrower or altered substrate specificity, whereas class Ia members with a conserved fold tend to be more promiscuous (McMahon et al., 2014; Santos‐Martin et al., 2021). BperDsbA also exhibits a negatively charged region below the catalytic site, a feature not typically observed in other DsbAs. These features likely underlie its restricted substrate range and adaptation to specific PTX‐related targets.

**FIGURE 6:**
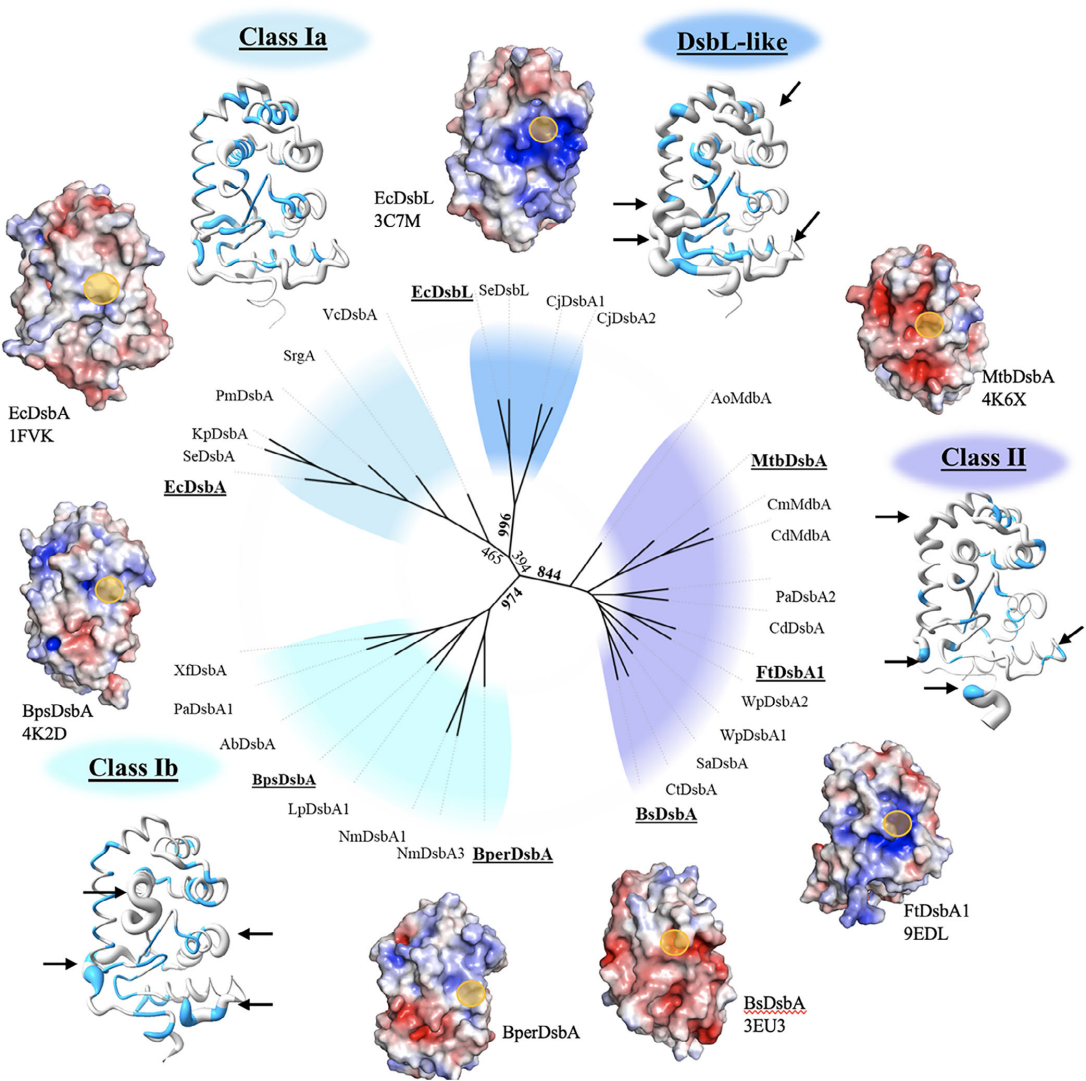
Phylogenetic and structural comparison of DsbA homologues.

Collectively, our findings demonstrate that BperDsbA, while structurally canonical, is functionally specialized among DsbA enzymes. Its highly oxidizing redox potential, distinct electrostatic profile, and narrow substrate specificity point to a dedicated role in the oxidative folding of virulence factors such as the pertussis toxin. To contextualize these properties, we carried out a comparative structural and phylogenetic analysis of 30 DsbA homologues (Figure 6), which resolved the family into four major clades: class Ia, Ib, II, and a DsbL‐like subgroup, each defined by conserved core features but divergent properties. These subclasses exhibit distinct structural and electrostatic traits, ranging from the broad, hydrophobic activity of class Ia enzymes to the charged, substrate‐focused architecture of class Ib and the specialized configurations of class II and DsbL‐like members, reflecting evolutionary adaptation to organism‐specific virulence functions. This clade‐specific variation underscores the evolutionary plasticity of the DsbA fold and highlights opportunities for developing pathogen‐specific anti‐virulence therapies. By exploiting these differences, it may be possible to design inhibitors that impair specific DsbA activity without disrupting DsbAs from beneficial bacteria, offering a promising alternative in the fight against antimicrobial resistance.

An unrooted neighbor‐joining consensus tree of 30 structurally characterized DsbA‐like proteins (Arts et al., 2013; Banaś et al., 2021; Christensen et al., 2016; Crow et al., 2009; Grimshaw et al., 2008; Guddat et al., 1997; Heras et al., 2008; Heras et al., 2010; Ireland et al., 2013; Kurth et al., 2013; Kurth et al., 2014; Kurz et al., 2009; Lafaye et al., 2009; Luong Truc et al., 2018; Martin et al., 1993; Penning et al., 2024; Premkumar et al., 2013; Premkumar et al., 2014; Reardon‐Robinson et al., 2015a; Reardon‐Robinson et al., 2015b; Shouldice et al., 2009; Walden et al., 2012; Walden et al., 2019) (see Table S1 for details). The tree resolves into four main clades—class Ia, Ib, II, and a DsbL‐like subgroup (bootstrap values separating clades are shown), broadly consistent with previously described groupings (McMahon et al., 2014; Totsika et al., 2018), with the addition of the DsbL‐like clade. Class Ia enzymes, including EcDsbA, exhibit high structural similarity, with minor variations in the N‐terminal region, inserted helical domain, and terminal loops. These proteins share a long hydrophobic groove near the active site and predominantly hydrophobic catalytic surfaces. Class Ib enzymes, including BperDsbA, show greater structural and electrostatic divergence, with distinct loop topologies, particularly in the region connecting the TRX domain to the inserted α‐helical domain and the loop preceding the final α‐helix. The latter helps shape a narrower peptide‐binding groove. These proteins, particularly BperDsbA, show bipolar surface charge distributions. Class II enzymes are the most structurally distinct, with rearranged β‐strands in the TRX core, unique loop insertions, and highly charged surface properties. The DsbL‐like clade, a subset of class Ia, is distinguished by intensely electropositive catalytic surfaces. Sequence and structural similarity to EcDsbA (PDB ID: 1FVK (Guddat et al., 1997; Martin et al., 1993)) is represented by blue shading (sequence identity) and cartoon thickness (structural deviation, RMSD). Black arrows denote regions with greater structural divergence. Catalytic cysteines are circled in yellow. Electrostatic surface potentials were calculated using APBS (Baker et al., 2001) in PyMOL (Delano, 2002), with positive and negative charges colored blue and red, respectively (±5 kT/e saturation). Structural superpositions and electrostatic comparisons were visualized in UCSF Chimera (Pettersen et al., 2004), and sequence identity and RMSD values were derived using TM‐align (Bittrich et al., 2024; Zhang & Skolnick, 2005).

## 4 MATERIALS AND METHODS

### 4.1 Cloning, expression, and purification

Unless stated otherwise LB media was used for bacterial growth supplemented where appropriate with 100 μg/mL of ampicillin or 34 μg/mL of chloramphenicol or both.

Codon optimized mature BperDsbA (locus SQE19771, signal peptide removed) with an N terminal TRX‐His_6_ tag was cloned into a pMCSG7 (Eschenfeldt et al., 2009) vector to create pMCSG7::His_6_‐TRX‐*BperDsbA* which was subsequently transformed into *E. coli* (C43) DE3 cells and expressed using an autoinduction method (Studier, 2005) (24 h at 30°C). Cells were harvested and resuspended in Tris buffer (20 mM Tris, 150 mM NaCl, 20 mM imidazole, pH 7) followed by sonication to lyse cells (Misonix S‐4000 ultrasonic Liquid Processor [Qsonica]). His_6_‐TRX tagged BperDsbA was purified from the resulting cytoplasmic extracts by nickel affinity chromatography followed by cleavage of the His_6_‐TRX tag by TEV protease and subsequently reverse immobilized metal affinity chromatography (IMAC). The cleaved protein was oxidized with 10 molar equivalents of oxidized glutathione (GSSG) (1–2 h at 4°C) and purified to homogeneity by size exclusion chromatography using a HiLoad® 16/600 Superdex® column (GE Healthcare) equilibrated in HEPES buffer (25 mM HEPES, 150 mM NaCl, pH 7).

For cell‐based assays, a second plasmid construct was utilized. The BperDsbA gene was cloned into pSU2718 that contained an EcDsbA signal peptide (EcDsbAss) (Hong et al., 2024), hereafter referred to as pSU2718‐BperDsbA. This allows the periplasmic expression of BperDsbA in *E. coli* K‐12 strains JCB817 (Δ*dsbA*) (Bardwell et al., 1991) and PL263 (MC4100 Δ*dsbC* Δ*mdoG*) (Leverrier et al., 2011) cells.

### 4.2 Validation of heterologous DsbA expression and in vivo oxidation state

To validate the functional expression of BperDsbA, EcDsbAss‐BperDsbA was subcloned with a C‐terminal hexahistidine tag into pDSW204 (Hong et al., 2024). Log phase cultures grown in LB and relevant antibiotics were harvested, washed with 1× PBS and used for whole cell, periplasmic extraction and AMS alkylation as previously described (Hong et al., 2024). In brief, the samples were resuspended in ice‐cold extraction buffer (20 mM Tris–HCl pH 8, 20% w/v sucrose, 1 mM EDTA, 1 mg/mL lysozyme). The supernatant collected is then precipitated with ice‐cold trichloroacetic acid at a final concentration of 20% v/v. For alkylation, the precipitated samples were resuspended in ice‐cold reaction buffer (50 mM Tris‐HCl, pH 7.5, 0.1% v/v SDS and 10 mM EDTA) and labeled with 20 mM AMS. An unlabeled negative control and a sample pretreated with 10 mM DTT prior to AMS treatment (positive alkylation control) were used as the experimental reference. The samples were separated on SDS‐PAGE under non‐reducing conditions and transferred to a PVDF membrane for Western blot analysis. The primary and secondary antibodies were used at the following dilutions: α‐His (1:500), α‐SurA (1:5000), α‐GroEL (1:50,000), and HRP‐conjugated α‐Rabbit IgG (1:1000).

### 4.3 Crystallization

BperDsbA crystals were produced via the hanging drop vapor diffusion method following 2 weeks of incubation at 293 K. Stacked, plate‐like crystals were produced in 2 μL droplets each containing 1 μL of 35 mg/mL protein and 1 μL of reservoir solution consisting of 0.1 M Bis‐Tris (pH 5.75) and 25% (w/v) PEG monomethyl ether 5000. Crystallization conditions were optimized from the 46th Index condition (Hampton Research, USA) (0.1 M BIS‐TRIS pH 6.5, 20% w/v PEG monomethyl ether 5000). Cryoprotectant consisting of 20% (v/v) glycerol was added directly to droplets containing crystals which were subsequently removed and cryocooled in liquid nitrogen prior to data collection.

### 4.4 Structural determination and refinement

BperDsbA diffraction data was collected at the Australian synchrotron using the MX2 beamline (13 keV, 100 K) equipped with an EIGER X 16 M pixel detector. Data was collected covering 360° with 0.1° per frame with 0.02 s exposure. The data was indexed and scaled using HKL2000 (Otwinowski & Minor, 1997), implementing a 1.65 Å cut‐off. Crystals belonged to the *C2* space group with cell dimensions of a ≈ 191.8 Å, b ≈ 37.2 Å, c ≈ 57.1 Å and α = 90.0°, β = 104.6° and γ = 90.0°, which was consistent with two molecules per asymmetric unit (Matthews, 1968). The crystal structure of BperDsbA was solved using molecular replacement (Phaser (Read, 2001)) with *B. parapertussis* DsbA (PDB ID: 3HD5) as a reference. The resulting model was refined using REFMAC5 (Murshudov et al., 2011) and Phenix (Afonine et al., 2010) and further built using COOT (Emsley et al., 2010). The quality of the model was monitored during refinement by the Rfree value, which represented 5% of the data. The structure was validated using the MolProbity server (Chen et al., 2010) and figures were created with PyMOL (Delano, 2002). Details of data‐processing statistics and final refinement values are summarized in Table 1.

### 4.5 Circular dichroism spectroscopy

Analysis of protein secondary structure and thermal stability was conducted using circular dichroism (CD) spectroscopy with a JASCO Model J‐1100 CD spectrophotometer (JASCO, USA). Both oxidized and reduced proteins were measured at 300 μg/mL prepared in 20 mM sodium phosphate, 0.1 mM EDTA, pH 7, with reduced and oxidized samples incubated overnight with 100 molar excesses of dithiothreitol (DTT) and GSSG respectively. Wavelength scans were conducted at 20°C while thermal unfolding was measured by monitoring CD millidegrees at 222 nm from 20°C to 90°C with a heating gradient of 0.5°C/min. Three replicates were taken for both oxidized and reduced proteins. The melting temperature was calculated according to the equation below, assuming a two‐state model using GraphPad Prism (GraphPad Software, Inc., San Diego, CA, USA):

$$Y=\begin{array}{l} \frac{\left(\left(y_{f}+\left(m_{f}*x\right)\right)-\left(y_{u}+\left(m_{u}*x\right)\right)\right)*\exp^{\left(\frac{h}{1.987*t}\right)*\left(\left(\frac{t}{\mathrm{Tm}}\right)-1\right)}}{\left(1+\exp^{\left(\frac{h}{1.987*t}\right)*\left(\left(\frac{t}{\mathrm{Tm}}\right)-1\right)}\right)}\\ +\;\left(y_{u}+\left(m_{u}*x\right)\right) \end{array}$$

where *Y* is CD signal (mdeg), *x* is temperature (°C), *t* is temperature *x* in Kelvin (K), Tm is the midpoint of the thermal unfolding curve (K), y_f_ is the intercept of the fully folded baseline pre‐transition, m_f_ is the gradient of the fully folded baseline pre‐transition, y_u_ is the intercept of the fully unfolded baseline post‐transition, m_u_ is the gradient of the fully unfolded baseline post‐transition, *h* is the change in enthalpy for unfolding at Tm (Pace et al., 1998).

### 4.6 Determination of redox potential

The redox potential of BperDsbA was measured by 4‐acetamide‐4′‐maleimidylstilbene‐2‐2′‐disulfonate (AMS) gel shift analysis as previously described (Inaba & Ito, 2002). AMS alkylates free thiols, adding ~0.5 kDa per cysteine, resulting in a 1 kDa shift when both catalytic cysteines of reduced BperDsbA are modified. Briefly, 5 μM BperDsbA was incubated overnight at room temperature in degassed buffer (100 mM sodium phosphate pH 7, 1 mM EDTA, 100 mM GSSG) supplemented with 10 μM to 1 mM reduced glutathione (GSH). Reactions were quenched with trichloroacetic acid (TCA), resuspended in AMS buffer (2 mM AMS, 50 mM Tris pH 7, 1% SDS), and analyzed by SDS‐PAGE. The relative amounts of oxidized and reduced BperDsbA were determined by densitometry (ImageJ: National Institutes of Health) and quantified as the ratio of reduced BperDsbA. The equilibrium constant (*K*_eq_) and redox potential were determined using the standard equations (Mössner et al., 1999). Briefly, the data were then fitted using GraphPad Prism (GraphPad Software, Inc., San Diego, CA, USA) and the equilibrium constant Keq estimated according to the equation below:

$$Y_{\mathrm{obs}}=\left(Y_{\mathrm{ox}}+\left(M/K_{\mathrm{eq}}\right)Y_{\mathrm{red}}\right)/1+\left(M/K_{\mathrm{eq}}\right)$$

where *Y*_obs_ is the fraction of reduced protein at equilibrium, *Y*_ox_ and *Y*_red_ are the signals for the oxidized and reduced proteins, respectively, and *M* is the ratio of [GSH]^2^/[GSSG].

The redox potential was determined from the Nernst equation:

$$E^{0^{\prime }}={E^{0^{\prime }}}_{\mathrm{GSH}/\text{GSSG}}–\left(\mathrm{RT}/\text{2F}\right)\times \ln\;K_{\mathrm{eq}}$$

where E^0′^_GSH/GSSG_ is the standard potential of −240 mV, *R* is the universal gas constant 8.314 J K^−1^ mol^−1^, *T* is the absolute temperature in K, F is the Faraday constant 9.648 × 10^4^ C mol^−1^, and *K*_eq_ is the equilibrium constant. *K*_eq_ was calculated as mean ± standard error of the mean (SEM) with three independent replicates.

### 4.7 In vitro thiol‐oxidoreductase activity

Thiol‐oxidase activity of BperDsbA was measured in vitro using a Fluorescence Resonance Energy Transfer (FRET) based assay with EcDsbL and Neisserial DsbA substrate‐derived peptides, ASST (**C**NENGL**C**K) (Lee et al., 2004) and PilQ (**C**QQGFDGTQNS**C**K) (Vivian et al., 2008), respectively. Oxidation of the cysteine pair in the peptides leads to cyclisation bringing the N‐terminal 1,4,7,10‐tetraazacyclododecane‐1,4,7,10‐tetraacetic acid (DOTA)—europium (Eu^3+^) group near the methylcoumarin‐labeled C‐terminal lysine, producing FRET upon excitation at 340 nm and emission at 615 nm.

Reactions were conducted in 50 μL volumes in 384‐well plates (PerkinElmer OptiPlate−384) containing 500 nM BperDsbA, 2 mM GSSG, and 15 μM peptide in MES buffer (50 mM MES, 50 mM NaCl, 5 mM EDTA, pH 5.5). Fluorescence (excitation *λ* = 340 nm and emission *λ* = 615 nm) was recorded over 30 min using a CLARIOstar plate reader (BMG Labtech) equipped with a TR‐FRET module. All measurements were performed in triplicate, and data are presented as mean ± SEM. Additionally a one‐way ANOVA was performed at the endpoint with either a Dunnett or Tukey's (as appropriate) post‐hoc against the buffer only control. All analyses were performed using GraphPad Prism 8 (GraphPad Software, San Diego, CA, USA).

In vitro Dsb reduction activity of BperDsbA was assessed using a previously described insulin reduction assay (Holmgren, 1979; Subedi et al., 2021). Briefly, the reaction mixture was prepared by mixing 131 μM bovine insulin and 10 μM BperDsbA in 100 mM sodium phosphate buffer (pH 7.0) supplemented with 2 mM EDTA and 0.35 mM DTT. Reduction of insulin's intramolecular Dsb results in precipitation of the B chain, which was monitored by measuring the increase in optical density at 650 nm over an 80‐min period. A one‐way ANOVA was performed with data at the 80 min timepoint with a Dunnett post‐hoc against the buffer‐only control. All analyses were performed using GraphPad Prism 8 (GraphPad Software).

A gel‐based approach was used to assess BperDsbA's ability to interact with a peptide derived from subunit 5 of the pertussis toxin (PTX) (QI**C**PLNGY**C**E) (Stenson & Weiss, 2002). In short, 50 μM of oxidized BperDsbA was incubated with 50 μM of PTX peptide for 5, 10 and 30 s in a solution of 100 mM sodium phosphate pH 7, 1 mM EDTA. Reactions were quenched and precipitated with TCA. The redox state of BperDsbA was subsequently analyzed by AMS alkylation and visualized by SDS‐PAGE as described above, with densitometry performed through the ImageJ programme (Schneider et al., 2012) to determine the proportion of reduced DsbA.

### 4.8 In vivo phosphatase oxidation assay

The ability of BperDsbA to interact with the *E. coli* DsbA substrate PhoA phosphatase was assessed in vivo using *E. coli* strain JCB817 carrying pWSK29‐PhoA. Cultures of the strains carrying pSU2718‐BperDsbA or other relevant controls were grown to mid‐log phase. PhoA and thiol oxidase expressions were then induced with 500 μM IPTG for 50 min, after which 1 mL samples were collected, adjusted to an OD₆₀₀ of 0.3, and treated with 100 μL of 1 M iodoacetamide to alkylate free thiols. Samples were incubated on ice for 20 min, then washed twice with wash buffer (200 mM Tris–HCl pH 7.3, 250 mM NaCl, 40 mM NH₄Cl) and resuspended in 1 mL of the same buffer.

For the activity assay, 100 μL aliquots of the resuspended cells were transferred into reaction tubes containing 12 μL chloroform, 12 μL 0.1% SDS, and 900 μL of reaction buffer (1 M Tris–HCl pH 8.0, 1 mM MgCl₂, 1 mM ZnCl₂). Samples were vortexed and incubated at 37°C for 5 min. Following incubation, 1.53 μL of 500 mM para‐nitrophenylphosphate (pNPP) was added, and tubes were mixed by inversion. Reactions were incubated at 28°C until a yellow color developed in the positive control (pSU2718‐EcDsbA). Reactions were stopped by adding 120 μL of stopping buffer (83.33 mM EDTA, 416.67 mM K₂HPO₄), and cell debris was removed by centrifugation at 20,000 × g for 3 min.

For analysis, 200 μL of the resulting supernatant was transferred to a 96‐well plate, and absorbance was measured at 420 and 550 nm. Phosphatase activity was calculated using the following equation:

$$\text{Units of activity}=1000\times \left[\left(A_{420}-1.75\times A_{550}\right)/\left(t\times v\times {\mathrm{OD}}_{600}\right)\right]$$

where *t* is the time (in minutes) taken for color development in the positive control, *v* is the initial volume of cells used (in mL), and OD₆₀₀ represents the optical density of the culture used in the assay.

Data are presented as the mean + SEM with a one‐way ANOVA performed with a Dunnett post‐hoc against the empty vector. All analyses were performed using GraphPad Prism 8 (GraphPad Software).

### 4.9 In vivo ASST oxidation assay

To determine whether BperDsbA can catalyze the oxidative folding of arylsulfate sulfotransferase (ASST) in vivo, ASST activity was monitored via a fluorescence‐based assay as previously described (Verderosa et al., 2021). In short, ASST‐expressing JCB817 (pWSK29‐ASST, IPTG inducible) transformants were grown overnight at 37°C with agitation in LB‐Lennox supplemented with antibiotics and 500 μM IPTG. Cultures were harvested, washed in 1× PBS and samples were subsequently normalized to an OD600 of 0.8 in PBS. In a 96‐well plate, 100 μL of normalized culture was diluted 1:1 with 1 mM potassium 4‐methylumbelliferyl sulphate (MUS) and 5 mM phenol and fluorescence (excitation *λ* = 340 nm and emission *λ* = 450 nm) was measured immediately using a CLARIOstar plate reader (BMG, Australia) over the course of 95 min to monitor ASST‐dependent substrate turnover. Data are presented as the mean + SEM with a one‐way ANOVA performed with a Dunnett post‐hoc against the empty vector. All analyses were performed using GraphPad Prism 8 (GraphPad Software).

### 4.10 In vivo FlgI oxidation assay

The ability of BperDsbA to oxidatively fold a component of the *E. coli* flagellar motor, FlgI (Hizukuri et al., 2006; Penning et al., 2024), was assessed in vivo. JCB817 strains carrying pSU2718‐BperDsbA or relevant controls were tested for bacterial swimming motility (indicative of thiol‐oxidase activity) in 24 well plates as previously described (Verderosa et al., 2021). Briefly, overnight cultures that had been incubated statically at 37°C were normalized to an OD_600_ of 0.6–0.74 and 1 μL of this culture was inoculated onto Nunc 24 plates containing 700 μL 0.25% motility LB agar supplemented with 500 μM IPTG and incubated at 37°C for 24 h. As bacteria migrate through the agar, a radius of motility corresponding to an increased OD_600_ was observed and monitored over the 24 h. incubation period using a CLARIOstar plate reader (BMG, Australia). Data are presented as the mean of five independent replicates ± SD, and analyzed by one‐way ANOVA using GraphPad Prism 8 (GraphPad Software).

### 4.11 Mucoidal morphology assay

To assess the in vivo disulphide reductase activity (indicative of isomerase activity) of BperDsbA, the production of colanic acid was assessed as previously described (Hong et al., 2024; Leverrier et al., 2011). In short, single isolates of transformed PL263 (∆*dsbC*, Δ*mdoG*) (Leverrier et al., 2011) cells were spotted onto 1.5% solid agar containing M9 media (1× M9 salts, 2 mM MgSO4, 100 μM CaCl_2_, 0.4% v/v glycerol, 0.1% w/v casamino acid) supplemented with IPTG (500 μM) and chloramphenicol (17 μg/mL). Following 48 h of incubation at 28°C, plates were imaged using a ChemiDoc MP Imaging System (Bio‐Rad) and visually inspected for changes in mucoidal morphology.

### 4.12 Structure‐based tree

A cladistic tree was generated to assess how the structure and sequence of BperDsbA compares to other structurally characterized DsbAs and to support our structural analyses. Thirty DsbA structures were retrieved from the PDB (Arts et al., 2013; Banaś et al., 2021; Christensen et al., 2016; Crow et al., 2009; Grimshaw et al., 2008; Guddat et al., 1997; Heras et al., 2008; Heras et al., 2010; Ireland et al., 2013; Kurth et al., 2013; Kurth et al., 2014; Kurz et al., 2009; Lafaye et al., 2009; Luong Truc et al., 2018; Martin et al., 1993; Penning et al., 2024; Premkumar et al., 2013; Premkumar et al., 2014; Reardon‐Robinson et al., 2015a; Reardon‐Robinson et al., 2015b; Shouldice et al., 2009; Walden et al., 2012; Walden et al., 2019) and were confirmed to be DsbA homologues by the presence of five α‐helices in the alpha helical insertion region, the presence of a *cis‐*proline loop and a five‐member β‐sheet in the TRX core. To create the tree, a multiple sequence alignment was performed utilizing the Expresso algorithm executed via the T‐coffee server (Taly et al., 2011) with the mature sequences of DsbA homologues deposited to the PDB. An unrooted neighbor‐joining consensus tree was then reconstructed from 1000 bootstrap replicates using the prodist, neighbor and consense programmes included in the PHYLIP package (Felsenstein, 1989). The tree was visualized using the Interactive Tree of Life (iTOL v7) web server (Letunic & Bork, 2024).

## AUTHOR CONTRIBUTIONS

**Stephanie Penning:** Investigation; writing – original draft; methodology; formal analysis; writing – review and editing; data curation; validation. **Lachlan Mitchell:** Investigation; writing – review and editing. **Yaoqin Hong:** Investigation; writing – review and editing; formal analysis; data curation. **Taylor Cunliffe:** Investigation; writing – review and editing. **Pramod Subedi:** Investigation; writing – review and editing. **Geqing Wang:** Investigation; writing – review and editing. **Lilian Hor:** Investigation; writing – review and editing. **Makrina Totsika:** Supervision; writing – review and editing; resources. **Jason J. Paxman:** Supervision; writing – review and editing; formal analysis; methodology. **Begoña Heras:** Conceptualization; funding acquisition; methodology; validation; writing – review and editing; project administration; supervision; resources.

## CONFLICT OF INTEREST STATEMENT

Makrina Totsika is an employee of the GSK group of companies. This research was conducted in the absence of any commercial or financial relationships that could be construed as a potential conflict of interest.

## Supporting information


**Figure S1.** BperDsbA oxidase activity against PilQ derived peptide. (A) Fluorescence curves measured at 650 nm indicates oxidation PilQ peptide by EcDsbA (open circles ○), BperDsbA (closed squares ■) or buffer control (open squares □). Only EcDsbA displayed PilQ oxidation while BperDsbA and the buffer control displayed similarly low levels of activity. Data represents one independent replicate with three technical replicates. (B) Motility was quantified on soft agar after 20 h at 37°C. Data are mean ± SEM of *n* = 5 biological replicates (EcDsbA, BperDsbA) or *n* = 4 (vector). Ordinary one‐way ANOVA revealed a significant effect of treatment (*F*(2, 11) = 1655, *p* < 0.0001, *R* ^2^ = 0.997). Tukey's multiple comparisons test showed that BperDsbA significantly increased motility relative to VC or vector control (mean difference = 0.2538, 95% CI = 0.1723–0.3352, *p* < 0.0001) but did not reach the level of EcDsbA (mean difference = 1.314, 95% CI = 1.237–1.390, *p* < 0.0001).
**Figure S2.** Statistical analyses of functional assays at endpoint. One‐way ANOVA analyses were used to determine the significance of BperDsbA's activity in (A) in vitro thiol oxidase activity, (B) in vitro disulphide reductase activity, (C) the swimming motility assay, and (D) in vivo activity against ASST. All graphs show mean ± SEM with individual points also shown. (A) BperDsbA showed significant activity against an ASST‐derived peptide after 28 min compared to the control (mean difference = −19,178, 95% CI = −31,210 to −7147, *p* < 0.05, *n* = 8). (B) BperDsbA displayed no detectable reductase activity after 80 min when compared to baseline measurements (mean difference = 0.001356, 95% CI = −0.02524 to 0.02795, *p* > 0.05, *n* = 3). (C) While BperDsbA transformed cells display limited motility over a 20 h incubation period, especially when compared to cells expressing EcDsbA, motility was significantly increased compared to an empty vector control (mean difference = 0.2538, 95% CI = 0.1723 to 0.3352, *p* < 0.0001, *n* = 5). (D) BperDsbA was unable to oxidize ASST in vivo, displaying a significant difference in comparison to the empty vector control (mean difference = −3531, 95% CI = −4651 to −2410, *p* < 0.0001, *n* = 4).
**Figure S3.** BperDsbA can be expressed heterologously. Periplasmic extracts from *E. coli* strain JCB817 expressing His‐tagged BperDsbA were analyzed by non‐reducing SDS–PAGE and immunoblotting using an anti‐His antibody. AMS alkylates free thiols, resulting in a detectable mass shift corresponding to slower migrating “reduced” species, whereas oxidized proteins lacking free thiols remain unshifted. A whole‐cell lysate of the JCB817 vector control (lane 1) was included as a negative control reference. Lane 2 shows the AMS‐treated periplasmic extract, lane 3 the untreated sample, and lane 4 the sample pre‐reduced with 10 mM DTT prior to AMS modification. Distinct oxidized and reduced forms of BperDsbA–His are indicated on the right. Immunoblotting with anti‐SurA confirmed periplasmic enrichment, while anti‐GroEL verified minimal cytoplasmic contamination.
**Figure S4.** In vitro oxidative activity of BperDsbA and EcDsbA against PTX derived peptides. (A) A second, representative replicate of BperDsbA and EcDsbA's activity against the QI**C**PLNGY**C**E peptide. BperDsbA becomes fully oxidized within seconds, whereas EcDsbA reaches only approximately 50% oxidation under the same conditions. (B) A representative replicate showing the activity of BperDsbA and EcDsbA against the SICNPGSSLC peptide. BperDsbA is less effective toward this substrate, reaching only ~50% oxidation within the 30‐s reaction window, while EcDsbA fails to meaningfully oxidize the peptide. The fraction of reduced protein was determined by densitometry using the ImageJ software package (Furniss et al., 2022).
**Figure S5.** Superimposition of BperDsbA monomers. Superimposition of BperDsbA monomers. Structural superimposition of the two monomers in the BperDsbA asymmetric unit (monomer A, blue; monomer B, gray) shows no significant conformational differences between the two.
**Figure S6.** Sequence and structural alignment of BperDsbA to homologues. Clustal Omega alignment (EMBL‐EBI) (left) and structural alignments (right) of BperDsbA (blue) were performed against of EcDsbA (PDB ID: 1FVK (Bardwell et al., 1993; Heras et al., 2009)) (light purple), BpsDsbA (PDB ID: 4K2D (Landeta et al., 2018)) (cyan) and PaDsbA1 (PDB ID: 3H93 (Inaba et al., 2006)) (dark purple). For the sequence alignment, identical residues (|) are shown in red, and similar residues (:) are shown in blue. The catalytic CXXC motif is highlighted in yellow, and the cis‐proline loop in green. BperDsbA shares 25% sequence identity with EcDsbA, aligning with an RMSD of 2.1 Å across 161 equivalent Cα atoms. It shares 39% sequence identity and an RMSD of 1.4 Å over 177 equivalent Cα atoms with BpsDsbA, and 38% sequence identity with an RMSD of 1.3 Å over 177 equivalent Cα atoms with PaDsbA1.
**Figure S7.** Conserved water coordination in EcDsbA and BperDsbA. Both EcDsbA (PDB ID: 1FVK (Bardwell et al., 1993; Heras et al., 2009)) and BperDsbA feature a highly acidic region on their non‐catalytic face which facilitates the coordination of water molecules within the core of the enzymes. The residues involved in this coordination and the relative positions of the water molecules are conserved across both DsbAs suggesting a shared proton relay mechanism (Bardwell et al., 1991). Atomic distances were calculated using PyMOL (Zapun et al., 1995), and the electrostatic potential was calculated with APBS (Rietsch et al., 1997) in PyMOL (Zapun et al., 1995) showing positive charges in blue (saturating at 5 kT/e) and negative charges in red (saturating at −5 kT/e).
**Figure S8.** Example of a Class II DsbA. Class II DsbAs differ from class I DsbAs in the topology of their core β‐sheet. While Class I DsbAs feature a topology of 3‐2‐4‐5‐1, Class II DsbAs, like BsDsbA (shown) (PDB ID: 3EU3 (Martin et al., 1993)) feature a topology of 1‐3‐2‐4‐5.
**Table S1.** Structurally characterized DsbA homologues.

## Data Availability

The data that support the findings of this study are available from the corresponding author upon reasonable request.
