# Supplementary material for: Structural and functional specialization of Bordetella pertussis DsbA for pertussis toxin folding
Source: Protein Sci. 2025 Dec 23;35(1):e70421. doi: 10.1002/pro.70421 (PMC12724015; doi:10.1002/pro.70421)
Supplement: Supplementary file 1 — Figure S1. BperDsbA oxidase activity against PilQ derived peptide. (A) Fluorescence curves measured at 650 nm indicates oxidation PilQ peptide by EcDsbA (open circles ○), BperDsbA (closed squares ■) or buffer control (open squares □). Only EcDsbA displayed PilQ oxidation while BperDsbA and the buffer control displayed similarly low levels of activity. Data represents one independent replicate with three technical replicates. (B) Motility was quantified on soft agar after 20 h at 37°C. Data are mean ± SEM of n = 5 biological replicates (EcDsbA, BperDsbA) or n = 4 (vector). Ordinary one‐way ANOVA revealed a significant effect of treatment (F(2, 11) = 1655, p < 0.0001, R 2 = 0.997). Tukey's multiple comparisons test showed that BperDsbA significantly increased motility relative to VC or vector control (mean difference = 0.2538, 95% CI = 0.1723–0.3352, p < 0.0001) but did not reach the level of EcDsbA (mean difference = 1.314, 95% CI = 1.237–1.390, p < 0.0001). Figure S2. Statistical analyses of functional assays at endpoint. One‐way ANOVA analyses were used to determine the significance of BperDsbA's activity in (A) in vitro thiol oxidase activity, (B) in vitro disulphide reductase activity, (C) the swimming motility assay, and (D) in vivo activity against ASST. All graphs show mean ± SEM with individual points also shown. (A) BperDsbA showed significant activity against an ASST‐derived peptide after 28 min compared to the control (mean difference = −19,178, 95% CI = −31,210 to −7147, p < 0.05, n = 8). (B) BperDsbA displayed no detectable reductase activity after 80 min when compared to baseline measurements (mean difference = 0.001356, 95% CI = −0.02524 to 0.02795, p > 0.05, n = 3). (C) While BperDsbA transformed cells display limited motility over a 20 h incubation period, especially when compared to cells expressing EcDsbA, motility was significantly increased compared to an empty vector control (mean difference = 0.2538, 95% CI = 0.1723 to 0.3352, p < 0.0001, n = 5 [file PRO-35-e70421-s001.docx]

**Supplementary Figures:**


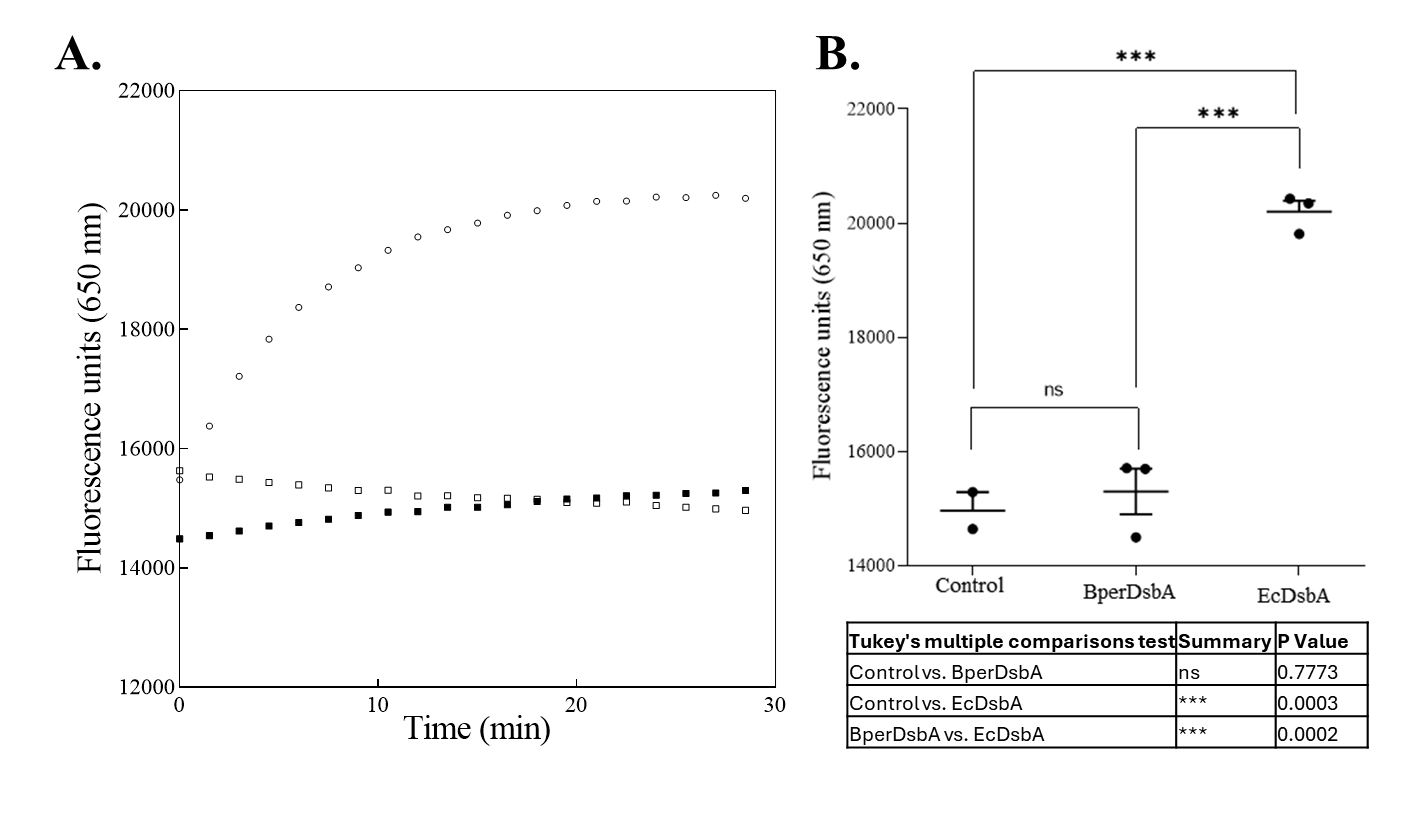


**Figure A1. BperDsbA oxidase activity against PilQ derived peptide.** **(A.)** Fluorescence curves measured at 650 nm indicates oxidation PilQ peptide by EcDsbA (open circles ○), BperDsbA (closed squares ■) or buffer control (open squares □). Only EcDsbA displayed PilQ oxidation while BperDsbA and the buffer control displayed similarly low levels of activity. Data represents one independent replicate with three technical replicates. **(B.)** Motility was quantified on soft agar after 20 h at 37 °C. Data are mean ± SEM of n = 5 biological replicates (EcDsbA, BperDsbA) or n = 4 (vector). Ordinary one-way ANOVA revealed a significant effect of treatment (F(2, 11) = 1655, p < 0.0001, R² = 0.997). Tukey’s multiple comparisons test showed that BperDsbA significantly increased motility relative to VC or vector control (mean difference = 0.2538, 95% CI = 0.1723–0.3352, p < 0.0001) but did not reach the level of EcDsbA (mean difference = 1.314, 95% CI = 1.237–1.390, p < 0.0001).


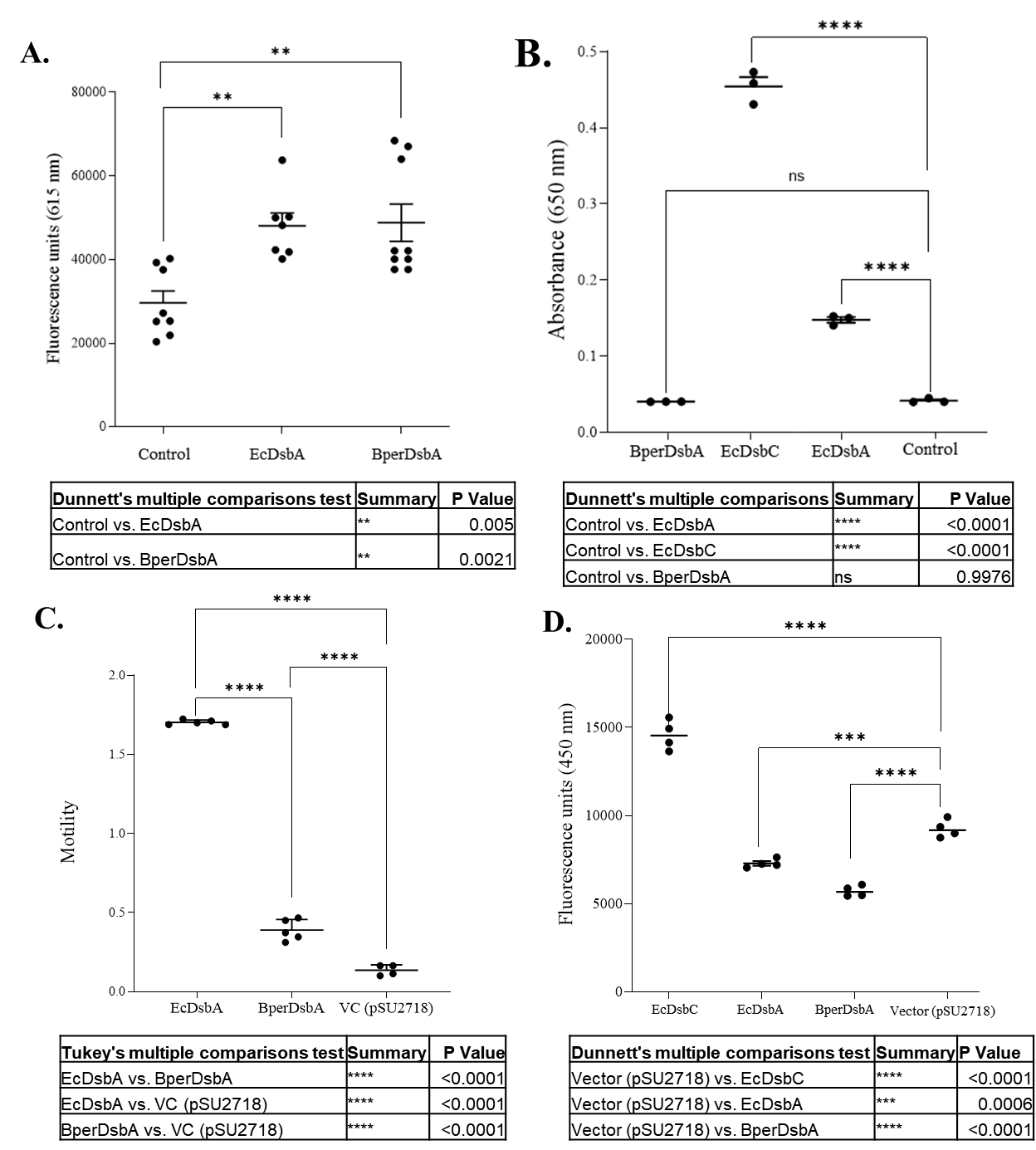


**Figure A2. Statistical Analyses of functional assays at endpoint.** One-way ANOVA analyses were used to determine the significance of BperDsbA’s activity in (**A.)** *in* *vitro* thiol oxidase activity, **(B.)** *in* *vitro* disulphide reductase activity, **(C.)** the swimming motility assay, and **(D.)** *in vivo* activity against ASST. All graphs show mean ± SEM with individual points also shown. **(A.)** BperDsbA showed significant activity against an ASST-derived peptide after 28 minutes compared to the control (mean difference = -19178, 95% CI= -31210 to -7147, p<0.05, n=8). **(B.)** BperDsbA displayed no detectable reductase activity after 80 minutes when compared to baseline measurements (mean difference = 0.001356, 95% CI= -0.02524 to 0.02795, p>0.05, n=3). **(C.)** While BperDsbA transformed cells display limited motility over a 20 h incubation period, especially when compared to cells expressing EcDsbA, motility was significantly increased compared to an empty vector control (mean difference = 0.2538, 95% CI= 0.1723 to 0.3352, p<0.0001, n=5). **(D.)** BperDsbA was unable to oxidise ASST *in vivo,* displaying a significant difference in comparison to the empty vector control (mean difference = -3531, 95% CI= -4651 to -2410, p<0.0001, n=4).


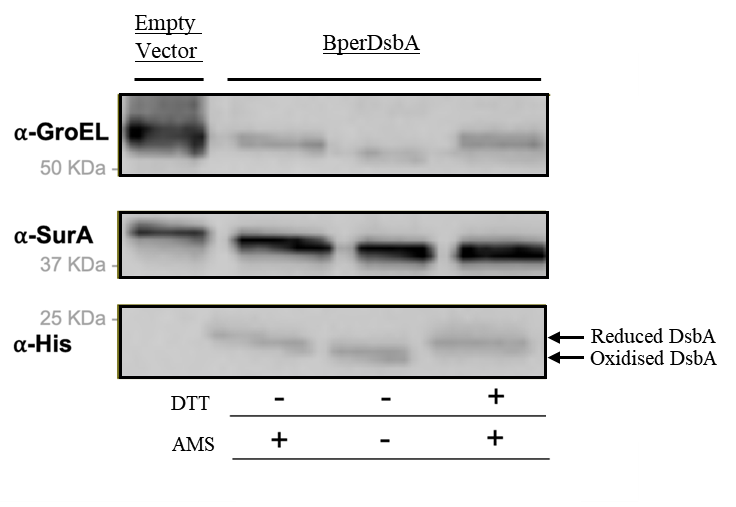


**Figure A3. BperDsbA can be expressed heterologously.** Periplasmic extracts from *E. coli* strain JCB817 expressing His-tagged BperDsbA were analysed by non-reducing SDS–PAGE and immunoblotting using an anti-His antibody. AMS alkylates free thiols, resulting in a detectable mass shift corresponding to slower migrating “reduced” species, whereas oxidised proteins lacking free thiols remain unshifted. A whole-cell lysate of the JCB817 vector control (lane 1) was included as a negative control reference. Lane 2 shows the AMS-treated periplasmic extract, lane 3 the untreated sample, and lane 4 the sample pre-reduced with 10 mM DTT prior to AMS modification. Distinct oxidised and reduced forms of BperDsbA–His are indicated on the right. Immunoblotting with anti-SurA confirmed periplasmic enrichment, while anti-GroEL verified minimal cytoplasmic contamination.


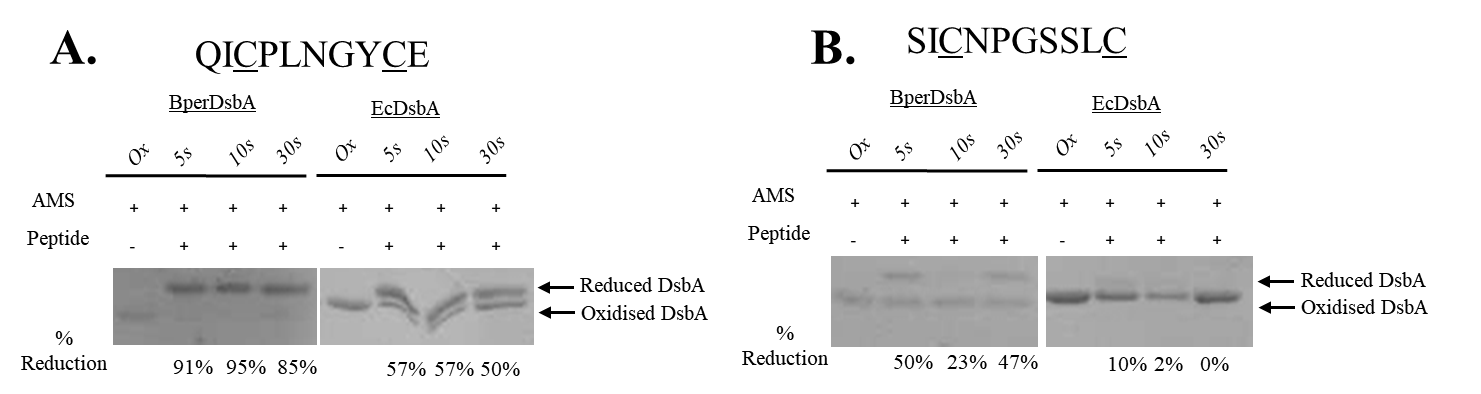


**Figure A4. *In vitro* oxidative activity of BperDsbA and EcDsbA against PTX derived peptides. (A.)** A second, representative replicate of BperDsbA and EcDsbA’s activity against the QICPLNGYCE peptide. BperDsbA becomes fully oxidised within seconds, whereas EcDsbA reaches only approximately 50% oxidation under the same conditions. **(B.)** A representative replicate showing the activity of BperDsbA and EcDsbA against the SICNPGSSLC peptide. BperDsbA is less effective toward this substrate, reaching only ~50% oxidation within the 30-second reaction window, while EcDsbA fails to meaningfully oxidise the peptide. The fraction of reduced protein was determined by densitometry using the ImageJ software package [1].


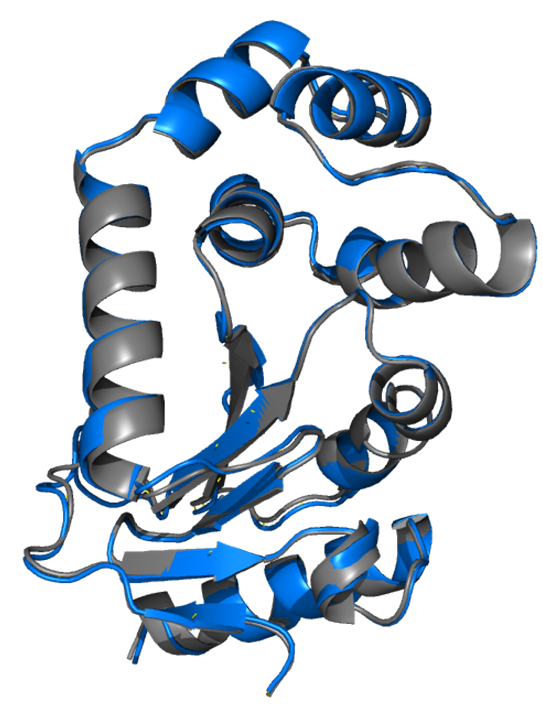


**Figure A5. Superimposition of BperDsbA monomers.** Superimposition of BperDsbA monomers. Structural superimposition of the two monomers in the BperDsbA asymmetric unit (monomer A, blue; monomer B, grey) shows no significant conformational differences between the two.


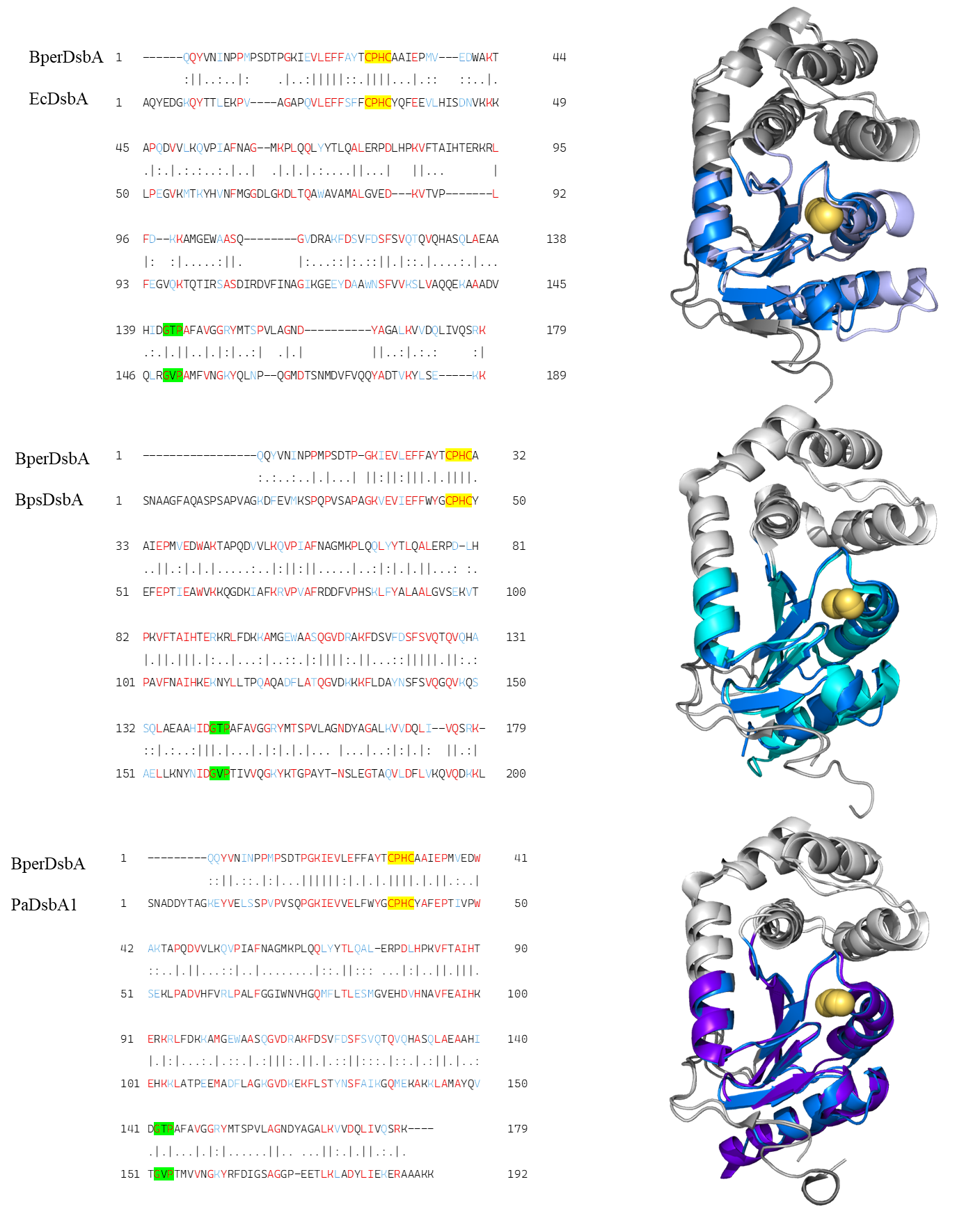


**Figure A6. Sequence and structural alignment of BperDsbA to homologues.** Clustal Omega alignment (EMBL-EBI) (left) and structural alignments (right) of BperDsbA (blue) were performed against of EcDsbA (PDB ID: 1FVK [2, 3]) (light purple), BpsDsbA (PDB ID :4K2D [4]) (cyan) and PaDsbA1 (PDB ID: 3H93 [5]) (dark purple). For the sequence alignment, identical residues (|) are shown in red, and similar residues (:) are shown in blue. The catalytic CXXC motif is highlighted in yellow, and the cis-proline loop in green. BperDsbA shares 25% sequence identity with EcDsbA, aligning with an RMSD of 2.1 Å across 161 equivalent Cα atoms. It shares 39% sequence identity and an RMSD of 1.4 Å over 177 equivalent Cα atoms with BpsDsbA, and 38% sequence identity with an RMSD of 1.3 Å over 177 equivalent Cα atoms with PaDsbA1.


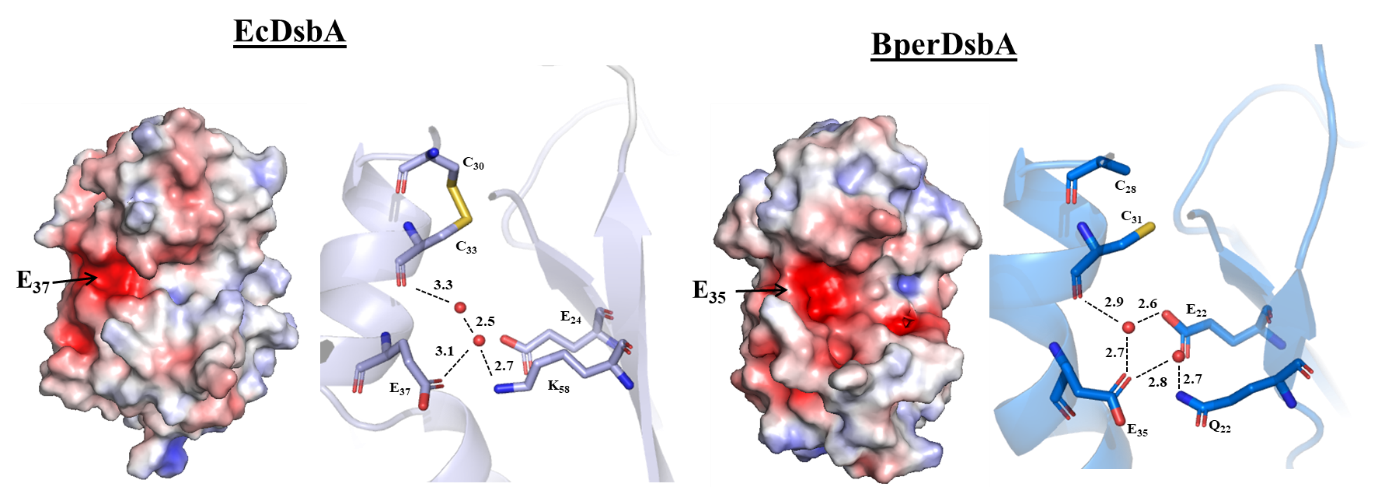


**Figure A7. Conserved water coordination in EcDsbA and BperDsbA.** Both EcDsbA (PDB ID: 1FVK [2, 3]) and BperDsbA feature a highly acidic region on their non-catalytic face which facilitates the coordination of water molecules within the core of the enzymes. The residues involved in this coordination and the relative positions of the water molecules are conserved across both DsbAs suggesting a shared proton relay mechanism [6]. Atomic distances were calculated using PyMOL [7], and the electrostatic potential was calculated with APBS [8] in PyMOL [7] showing positive charges in blue (saturating at 5 kT/e) and negative charges in red (saturating at -5 kT/e).

**
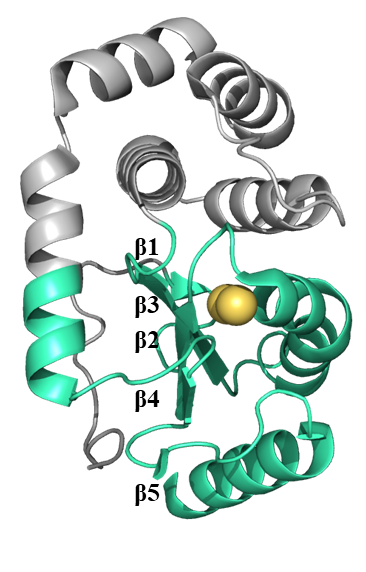
**

**Fig A8. Example of a Class II DsbA.** Class II DsbAs differ from class I DsbAs in the topology of their core β-sheet. While Class I DsbAs feature a topology of 3-2-4-5-1, Class II DsbAs, like BsDsbA (shown) (PDB ID: 3EU3 [9]) feature a topology of 1-3-2-4-5.

**Table A1. Structurally characterised DsbA homologues**

| **Protein name** | **PDB ID** | **Reference** | **Sequence Identity to EcDsbA (%)** | **RMSD to EcDsbA (Å)** | **TM score** | **Class** |
| --- | --- | --- | --- | --- | --- | --- |
| **EcDsbA** | 1FVK | [2, 3] |  |  |  | Ia |
| **SeDsbA** | 3L9S | [10] | 85 | 1.02 | 0.95 | Ia |
| **KpDsbA** | 4MCU | [11] | 82 | 1.2 | 0.96 | Ia |
| **PmDsbA** | 4OCE | [12] | 57 | 1.17 | 0.95 | Ia |
| **VcDsbA** | 4DVC | [13] | 40 | 1.75 | 0.86 | Ia |
| **SrgA** | 3L9V | [10] | 35 | 1.45 | 0.9 | Ia |
| **EcDsbL** | 3C7M | [14] | 25 | 2.23 | 0.8 | L |
| **CjDsbA1** | 7PQ7 | [15] | 25 | 2.63 | 0.75 | L |
| **CjDsbA2** | 7PQF | [15] | 28 | 2.55 | 0.77 | L |
| **SeDsbL** | 3L9U | [10] | 26 | 2.3 | 0.8 | L |
| **PaDsbA1** | 3H93 | [5] | 31 | 2.27 | 0.81 | Ib |
| **AbDsbA** | 4P3Y | [16] | 30 | 2.36 | 0.78 | Ib |
| **LpDsbA1** | 4JRR | Not yet published | 26 | 2.35 | 0.8 | Ib |
| **BpsDsbA** | 4K2D | [4] | 26 | 2.56 | 0.81 | Ib |
| **NmDsbA1** | 3DVW | [17] | 21 | 2.34 | 0.79 | Ib |
| **NmDsbA3** | 3DVX | [17] | 21 | 2.76 | 0.77 | Ib |
| **BperDsbA** | 9PH2 | This study | 20 | 2.16 | 0.67 | Ib |
| **XfDsbA** | 2REM | Not yet published | 19 | 2.46 | 0.8 | Ib |
| **BsDsbA** | 3EU3 | [9] | 18 | 3.3 | 0.68 | II |
| **CdDsbA** | 4PWO | Not yet published | 18 | 3.76 | 0.66 | II |
| **MtbDsbA** | 4K6X | [18] | 17 | 3.71 | 0.61 | II |
| **CmMdbA** | 6BO0 | [19] | 16 | 3.19 | 0.63 | II |
| **SaDsbA** | 3BCI | [20] | 14 | 3.1 | 0.66 | II |
| **CdMdbA** | 5C00 | [21] | 14 | 3.08 | 0.64 | II |
| **AoMdbA** | 4Z7X | [22] | 13 | 3.9 | 0.62 | II |
| **PaDsbA2** | 4N30 | [23] | 12 | 3.26 | 0.64 | II |
| **WpDsbA2** | 6EEZ | [24] | 11 | 2.91 | 0.7 | II |
| **FtDsbA1** | 9EDL | [25] | 11 | 3.57 | 0.65 | II |
| **WpDsbA1** | 3F4R | [26] | 10 | 3.55 | 0.64 | II |
| **CtDsbA** | 5KBC | [27] | 10 | 3.62 | 0.62 | II |

**References**

1. Schneider, C.A., W.S. Rasband, and K.W. Eliceiri, *NIH Image to ImageJ: 25 years of image analysis.* Nature Methods, 2012. **9**(7): p. 671-675.

2. Martin, J.L., J.C.A. Bardwell, and J. Kuriyan, *Crystal structure of the DsbA protein required for disulphide bond formation in vivo.* Nature, 1993. **365**(6445): p. 464-468.

3. Guddat, L.W., et al., *Structural analysis of three His32 mutants of DsbA: Support for an electrostatic role of His32 in DsbA stability.* Protein Science, 1997. **6**(9): p. 1893-1900.

4. Ireland, P.M., et al., *Disarming Burkholderia pseudomallei: Structural and Functional Characterization of a Disulfide Oxidoreductase (DsbA) Required for Virulence In Vivo.* Antioxidants & Redox Signaling, 2013. **20**(4): p. 606-617.

5. Shouldice, S.R., et al., *Characterization of the DsbA Oxidative Folding Catalyst from Pseudomonas aeruginosa Reveals a Highly Oxidizing Protein that Binds Small Molecules.* Antioxidants & Redox Signaling, 2009. **12**(8): p. 921-931.

6. Wang, G., et al., *A Buried Water Network Modulates the Activity of the Escherichia coli Disulphide Catalyst DsbA.* Antioxidants (Basel, Switzerland), 2023. **12**(2): p. 380.

7. Delano, W.L., *The PyMOL Molecular Graphics System.* <https://www.pymol.org>, 2002.

8. Baker, N.A., et al., *Electrostatics of nanosystems: application to microtubules and the ribosome.* Proc Natl Acad Sci U S A, 2001. **98**(18): p. 10037-41.

9. Crow, A., et al., *Crystal Structure and Biophysical Properties of Bacillus subtilis BdbD: AN OXIDIZING THIOL:DISULFIDE OXIDOREDUCTASE CONTAINING A NOVEL METAL SITE*.* Journal of Biological Chemistry, 2009. **284**(35): p. 23719-23733.

10. Heras, B., et al., *Structural and Functional Characterization of Three DsbA Paralogues from Salmonella enterica Serovar Typhimurium*.* Journal of Biological Chemistry, 2010. **285**(24): p. 18423-18432.

11. Kurth, F., et al., *Comparative sequence, structure and redox analyses of Klebsiella pneumoniae DsbA show that anti-virulence target DsbA enzymes fall into distinct classes.* PLoS One, 2013. **8**(11): p. e80210.

12. Kurth, F., et al., *Crystal structure of the dithiol oxidase DsbA enzyme from proteus mirabilis bound non-covalently to an active site peptide ligand.* J Biol Chem, 2014. **289**(28): p. 19810-22.

13. Walden, P.M., et al., *The 1.2 A resolution crystal structure of TcpG, the Vibrio cholerae DsbA disulfide-forming protein required for pilus and cholera-toxin production.* Acta Crystallographica Section D, 2012. **68**(10): p. 1290-1302.

14. Grimshaw, J.P.A., et al., *DsbL and DsbI Form a Specific Dithiol Oxidase System for Periplasmic Arylsulfate Sulfotransferase in Uropathogenic Escherichia coli.* Journal of Molecular Biology, 2008. **380**(4): p. 667-680.

15. Banaś, A.M., et al. *Interplay between DsbA1, DsbA2 and C8J_1298 Periplasmic Oxidoreductases of Campylobacter jejuni and Their Impact on Bacterial Physiology and Pathogenesis*. International Journal of Molecular Sciences, 2021. **22**, DOI: 10.3390/ijms222413451.

16. Premkumar, L., et al., *Structure of the Acinetobacter baumannii Dithiol Oxidase DsbA Bound to Elongation Factor EF-Tu Reveals a Novel Protein Interaction Site.* Journal of Biological Chemistry, 2014. **289**(29): p. 19869-19880.

17. Lafaye, C., et al., *Biochemical and Structural Study of the Homologues of the Thiol–Disulfide Oxidoreductase DsbA in Neisseria meningitidis.* Journal of Molecular Biology, 2009. **392**(4): p. 952-966.

18. Premkumar, L., et al., *Rv2969c, essential for optimal growth in Mycobacterium tuberculosis, is a DsbA-like enzyme that interacts with VKOR-derived peptides and has atypical features of DsbA-like disulfide oxidases.* Acta Cryst. D, 2013. **69**(10): p. 1981-1994.

19. Luong Truc, T., et al., *Structural Basis of a Thiol-Disulfide Oxidoreductase in the Hedgehog-Forming Actinobacterium Corynebacterium matruchotii.* Journal of Bacteriology, 2018. **200**(9): p. 10.1128/jb.00783-17.

20. Heras, B., et al., *Staphylococcus aureus DsbA Does Not Have a Destabilizing Disulfide: A NEW PARADIGM FOR BACTERIAL OXIDATIVE FOLDING*.* Journal of Biological Chemistry, 2008. **283**(7): p. 4261-4271.

21. Reardon-Robinson, M.E., et al., *A thiol-disulfide oxidoreductase of the Gram-positive pathogen Corynebacterium diphtheriae is essential for viability, pilus assembly, toxin production and virulence.* Molecular Microbiology, 2015. **98**(6): p. 1037-1050.

22. Reardon-Robinson, M.E., et al., *A Disulfide Bond-forming Machine Is Linked to the Sortase-mediated Pilus Assembly Pathway in the Gram-positive Bacterium Actinomyces oris*.* Journal of Biological Chemistry, 2015. **290**(35): p. 21393-21405.

23. Arts, I.S., et al., *Dissecting the Machinery That Introduces Disulfide Bonds in Pseudomonas aeruginosa.* mBio, 2013. **4**(6): p. e00912-13.

24. Walden, P.M., et al., *The atypical thiol-disulfide exchange protein [alpha]-DsbA2 from Wolbachia pipientis is a homotrimeric disulfide isomerase.* Acta Crystallographica Section D, 2019. **75**(3): p. 283-295.

25. Penning, S., et al., *Unveiling the versatility of the thioredoxin framework: Insights from the structural examination of Francisella tularensis DsbA1.* Computational and Structural Biotechnology Journal, 2024. **23**: p. 4324-4336.

26. Kurz, M., et al., *Structural and Functional Characterization of the Oxidoreductase α-DsbA1 from Wolbachia pipientis.* Antioxidants & Redox Signaling, 2009. **11**(7): p. 1485-1500.

27. Christensen, S., et al., *Structural and Biochemical Characterization of Chlamydia trachomatis DsbA Reveals a Cysteine-Rich and Weakly Oxidising Oxidoreductase.* PLOS ONE, 2016. **11**(12): p. e0168485.
